# Retrogene Duplication and Expression Patterns Shaped by the Evolution of Sex Chromosomes in Malaria Mosquitoes

**DOI:** 10.3390/genes13060968

**Published:** 2022-05-28

**Authors:** Duncan Miller, Jianhai Chen, Jiangtao Liang, Esther Betrán, Manyuan Long, Igor V. Sharakhov

**Affiliations:** 1 Department of Entomology, Virginia Polytechnic Institute and State University, Blacksburg, VA 24061, USA; duncanmiller@vt.edu (D.M.); jtliang@vt.edu (J.L.); 2 Department of Ecology and Evolution, University of Chicago, Chicago, IL 60637, USA; jianhaichen@uchicago.edu; 3 Department of Biology, University of Texas at Arlington, Arlington, TX 76019, USA; betran@uta.edu; 4 Department of Genetics and Cell Biology, Tomsk State University, 634050 Tomsk, Russia

**Keywords:** *Anopheles coluzzii*, *An. gambiae*, malaria mosquitoes, male-biased expression, meiotic sex chromosome inactivation, retrogene, retroposition, sexual antagonism, sex chromosome evolution

## Abstract

Genes that originate during evolution are an important source of novel biological functions. Retrogenes are functional copies of genes produced by retroduplication and as such are located in different genomic positions. To investigate retroposition patterns and retrogene expression, we computationally identified interchromosomal retroduplication events in nine portions of the phylogenetic history of malaria mosquitoes, making use of species that do or do not have classical sex chromosomes to test the roles of sex-linkage. We found 40 interchromosomal events and a significant excess of retroduplications from the X chromosome to autosomes among a set of young retrogenes. These young retroposition events occurred within the last 100 million years in lineages where all species possessed differentiated sex chromosomes. An analysis of available microarray and RNA-seq expression data for *Anopheles gambiae* showed that many of the young retrogenes evolved male-biased expression in the reproductive organs. Young autosomal retrogenes with increased meiotic or postmeiotic expression in the testes tend to be male biased. In contrast, older retrogenes, i.e., in lineages with undifferentiated sex chromosomes, do not show this particular chromosomal bias and are enriched for female-biased expression in reproductive organs. Our reverse-transcription PCR data indicates that most of the youngest retrogenes, which originated within the last 47.6 million years in the subgenus *Cellia*, evolved non-uniform expression patterns across body parts in the males and females of *An. coluzzii*. Finally, gene annotation revealed that mitochondrial function is a prominent feature of the young autosomal retrogenes. We conclude that mRNA-mediated gene duplication has produced a set of genes that contribute to mosquito reproductive functions and that different biases are revealed after the sex chromosomes evolve. Overall, these results suggest potential roles for the evolution of meiotic sex chromosome inactivation in males and of sexually antagonistic conflict related to mitochondrial energy function as the main selective pressures for X-to-autosome gene reduplication and testis-biased expression in these mosquito lineages.

## 1. Introduction

Gene retroposition is a mechanism of gene duplication and an important driver of organismal evolution. Retroduplications occur by means of reverse transcription of an mRNA template from a parental gene into a strand of complementary DNA (cDNA) using the enzymatic machinery encoded by retrotransposons, which are parasitic elements commonly occurring in eukaryotic genomes [1]. The newly synthesized cDNA, referred to as a retrocopy, is inserted into the genome at a new location [2]. As a result of being a product of mRNA, neither introns nor the regulatory sequences are part of the retrocopies. Intron loss is the feature of gene retroposition that has been used to identify retrocopies in diverse organisms [3,4,5,6,7]. Most retrocopies become retropseudogenes as a result of not having the necessary regulatory sequences that a gene requires to be properly expressed [1]. Very few retroduplications result in the formation of a transcriptionally active retrogene. This is due to the low chance that a retrocopy will be inserted into a genomic location where it can be expressed, remain functional and become fixed in a population. If a gene provides a cost in fitness or even if it is just neutral, it will likely not become fixed [1]. 

Some retrogenes have attributes that seem to facilitate their spread and fixation in a species. Two trends have been commonly observed. First, there is a predisposition for parental genes (original single-copy genes) to be X-linked and for retrogenes (duplicates) to be located on autosomes in organisms with heteromorphic sex chromosomes, including mammals and *Drosophila* [8,9,10,11]. The second trend is that autosomal retrogenes formed from parental genes on the X chromosome often exhibit male-biased expression [11,12,13]. The selective pressures that favor retrogenes from parental genes on the X chromosome are still not entirely understood and might be diverse. A number of factors likely contribute to these trends, including: sexual antagonism [14,15,16,17] and chromatin environment, including meiotic sex chromosome inactivation (MSCI) [8,9,18]. MSCI is an observed epigenetic phenomenon in heterogametic sexes in mammals where, during male meiosis, unpaired dimorphic chromosomes become transcriptionally silenced [19,20]. Intralocus sexual antagonism is the phenomenon where males and females have different fitness for different allelic variants of a gene. A shared genome causes limitations in fitness because different alleles at a given locus are favored by selection in males and females, and this antagonism cannot be resolved. It has been proposed that gene duplications that contribute to sexual dimorphism and tend to foster male-biased expression are the product of the resolution of this type of conflict [15,16,17]. Although we might not be certain of the selective pressures unless we catch the system as it is evolving, the duplication patterns and functional examples support these hypotheses. In *Drosophila melanogaster*, for example, a ~200,000-year-old gene duplication resulted in the evolution of essential functions specific to each sex, but also resulted in the downregulation of the antagonistic gene in the opposite sex [14]. 

Most investigations of retrogenes have focused on mammals and fruit flies [11] or other well-studied organisms [21], so there is a gap in functional studies of retrocopies in non-model species of eukaryotes. In particular, figuring out how retrogenes originate, evolve, and function could be important for understanding reproductive function in human disease vectors such as malaria mosquitoes, which are responsible for thousands of human deaths each year. Across the world, mosquitoes of the genus *Anopheles* act as vectors for the transmission of malaria parasites [22]. Developing new population control methods for these vectors is essential as mosquitoes are becoming increasingly resistant to current methods of population control based on insecticide treatment [23]. Research into the role of retrogenes in spermatogenesis could be a source for novel mosquito control strategies by exploiting the reproductive biology of these disease vectors. Understanding the genes involved in mosquito reproduction could improve future genetic techniques to reduce or prevent them from reproducing.

In addition, *Anopheles* mosquitoes have heteromorphic sex chromosomes, whereas *Aedes* and *Culex* mosquitoes have homomorphic sex chromosomes [24]. One investigation of gene retrocopies in *An. gambiae* and *Aedes aegypti* showed 400% more duplications than expected of genes from the X chromosome to another chromosome (i.e., X-to-autosome) in the *An. gambiae* lineage after the split with *Ae. aegypti* [25]. The study concluded that the acquisition of heteromorphic sex chromosomes that evolved in *Anopheles* after the divergence of the *Anopheles* and *Aedes* lineage from their ancestor with homomorphic sex chromosomes contributed to this pattern. Another study, using a more stringent dataset, found a 53% excess of X-to-autosome duplication events within the *Anopheles* lineage [26]. Moreover, retrogenes had a higher incidence of tissue-specific gene expression and testis-specific genes were depleted on the *Anopheles* X chromosome. The study also found that testis-biased expression is greater for retrogenes in the X-to-autosome duplication category compared to retrogenes originating from autosome-to-autosome duplication events [26]. A genomic comparison between *D. melanogaster, Ae. aegypti* and *Culex quinquefasciatus* found a relative paucity of genes with male-biased expression on the *Anopheles* X chromosome, and it has been argued that the X-to-autosome retroposition of genes that evolve male-biased expression could contribute to the evolution of that paucity, which is also called X chromosome demasculinization [27]. However, there is still a considerable lack of knowledge about factors that facilitated the evolution of these patterns. An RNA-seq expression study of *An. gambiae* cell populations from multiple stages of spermatogenesis provided evidence for the presence of MSCI from the transcriptional silencing of the X chromosome during the meiotic and postmeiotic phases [28]. MSCI could generate selection for increased dosage and favor the fixation of autosomal retrogenes potentially beneficial for male gametogenesis in mosquitoes. When a retroduplication of a parental gene from the X chromosome to an autosome occurs, the new retrogene can be expressed during meiosis since it is not affected by MSCI in the heterogametic sex [19,20]. It is also possible that autosomal retrogenes can resolve intralocus sexual conflict by providing specific functions to both sexes (a male-biased and a female-biased gene) [29,30] or to males (a somatic gene and a male-germline-biased gene) [17]. 

To understand the duplication patterns of retrogenes in malaria mosquitoes and to further test those predictions, this study used comparisons between retrogene and parental gene pairs and between chromosomal locations of retrogenes of different ages to see how they correlated with sex chromosome evolution. We used available expression data in combination with our reverse-transcription PCR (RT-PCR) experiments to further characterize the expression patterns and possible functions of retrogenes in *Anopheles*.

## 2. Materials and Methods

### 2.1. Identifying and Dating Gene Retroposition Events 

**Figure 1:**
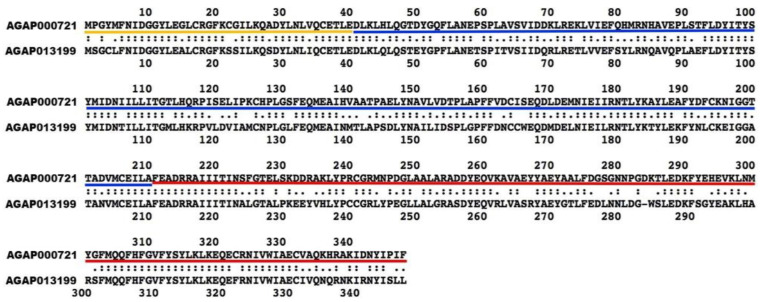
Example of a protein alignment for a parental gene and a retrogene. The parental gene (**top**) is AGAP000721 and the retrogene (**bottom**) is AGAP013199. Yellow, blue, and red lines indicate first, second, and third exons of the parental gene, respectively.

To identify retrogene and parental gene pairs, we used the gene annotation datasets for 19 species of *Anopheles*, *Aedes aegypti, Culex quinquefasciatus*, and *Drosophila melanogaster* (Table S1). Retrogene and parental gene pairs were identified computationally following the approaches described previously [8,9]. Briefly, protein BLAST was used to screen for paralogous gene pairs in the *An. gambiae* PEST (AgamP4) genome [31] based on all transcripts. The FASTA36 program was used to manually check the loss of candidate introns in retrogene sequences [32] (Supplementary file S1). In the case of a parental gene with multiple short introns, which may indicate mis-assignment of parental-derived status, we required more conserved syntenic neighbors in the parental gene than the retrogene. We also required that the alignment-length coverage between retrogenes and parental genes be reciprocally >70%, that exon-junction points of retrogenes cover ≥ 30 base pairs (10 amino acids) in both directions and that retrogene and parental gene be on different chromosomes. To capture more ancient retrogenes while controlling false discovery, we kept retrogene and parental gene pairs with a protein identify >30%. For example, the protein alignment for retrogene AGAP013199 and its parental gene, AGAP000721, revealed three exons in the parent gene and a single exon in the retrogene (Figure 1). The dating of the retroduplication event was achieved by a gene synteny-based method [33]. The gene age was assigned based on the most distant occurrence of gene synteny, i.e., at least two genes with a conserved gene order. Each retroduplication was assigned to a phylogenetic stage defined as a lineage having a previously estimated divergence time [34,35,36]. Twelve *Drosophila* species genomes [37] were used as the outgroups to exclude the possibility of synteny loss in distant species. The directions of gene duplication “from an autosome to a different autosome”, “from an autosome to the X chromosome”, and “from the X chromosome to an autosome” were noted as “A → A”, “A → X”, “X → A”, respectively. In lineages where there were no differentiated sex chromosomes, we still referred to “A → A”, “A → X”, “X → A”, and we call X the homolog of the chromosome that evolved to be the X chromosome in the closely related/heteromorphic sex chromosomes lineage. Since chromosome arm 1p in *Aedes* corresponds to the X chromosome in *Anopheles* [38], most genes on the X have conserved gene synteny on homomorphic chromosome 1. Whole chromosome genome assemblies were used as chromosome proxies. The synteny conservation of retrogenes and parental genes was used to trace the earliest branch of conserved gene synteny. The expected frequencies of the three types of movements were determined with the method of Betrán et al. (2002) [8].

### 2.2. Microarray and RNA-Seq Data

The expression of retrogenes and parental genes was analyzed based on three datasets: microarray data for sex-tissues [39], bulk RNA-seq data for sex-tissues [40], and RNA-seq data for cell-lineages of spermatogenesis stages [28]. For microarray data, *An. gambiae* sex-tissues of testes and ovaries were analyzed [39] and expression levels were recorded as mean log_2_ Robust Multi-array Average (RMA) values. For the bulk RNA-seq data of *An. gambiae*, sex-tissues including male reproductive organs (consisted of testis and accessory glands) and female reproductive organs (consisted of ovaries and common oviduct) were analyzed and the expression levels were normalized with mean transcript abundance in transcripts per million (TPM + 10^−3^). Both microarray and RNA-seq raw data were downloaded from VectorBase (https://vectorbase.org; February 2022; [41]). The raw data of germline stages of spermatogenesis RNA-seq expression was retrieved from [28] and normalized here with ln(FPKM + 1), which is the average of the natural logarithmic treatment of FPKM expression values. We defined sex-biased and unbiased genes based on whether significantly different expression (two-tailed Student’s *t*-test, *p* < 0.05) was detected between male reproductive organs (testes) and female reproductive organs (ovaries). We considered the expression male-biased if a gene were expressed significantly higher in testes than in ovaries [39,40].

### 2.3. Mosquito Strain and Dissection

The *An. coluzzii* MOPTI strain was used for wet-lab experiments. The mosquito colony was originally isolated from a population in N’Gabacoro Droit, Mali, West Africa in 1995. Mosquitoes were raised in a lab insectary by feeding larvae with fish food and adults with 1% sugar water. Females were fed defibrinated sheep blood (Colorado Serum Co., Denver, Colorado, USA) by means of artificial blood feeders to stimulate egg development. Virgin one-to-two-day old adult male and female mosquitoes were dissected. Heads, thoraxes, abdomens, and reproductive organs were separated. Testis and male accessory glands (MAGs) were dissected as well. Dissection of gonads was performed by removal of the last three somites of the mosquito abdomen. A total of 20 mosquitoes were dissected for both males and females. We dissected separately testes and MAGs from the same 20 males, which were 0–12 h-old virgin adults.

### 2.4. Primer Design, RNA Extraction, and cDNA Synthesis for RT-PCR

Primers for RT-PCR were designed using the NCBI Primer Blast tool [42]. All primer pairs (Table S2) were designed after comparing retrogene and parental gene sequences, as well as any paralog sequences to minimize any non-specific amplification. Dissected tissues were used for RNA extraction with the Direct-Zol^TM^ RNA MiniPrep Kit (Zymo Research, Irvine, CA, USA) following the provided protocol. The RNA was analyzed using nano-drop spectrophotometry to ensure the quality of the RNA extraction. The RNA concentrations were 5.4 ng/μL for testes from 20 adults and 18.2 ng/μL for MAGs from 20 adults. Extracted RNA was reverse transcribed into cDNA using the SuperScript™ III First-Strand Synthesis System (Thermo Fisher Scientific, Waltham, MA, USA) following the manufacturer’s protocol. 

### 2.5. Reverse-Transcription PCR

Synthesized cDNA was used to conduct two-step reverse-transcription PCR (RT-PCR). Each 20 µL RT-PCR reaction was performed using 10 µL of Platinum^TM^ Hot Start PCR Master Mix (Thermo Fisher Scientific, Vilnius, Lithuania), 8.5 µL of ddH_2_O, 0.5 µL forward primer, 0.5 µL reverse primer, and 0.5 µL of cDNA in a C1000 Touch^TM^ Thermal Cycler (Bio-Rad, Hercules, CA, USA). We used the following temperatures for every PCR reaction cycle: 94 °C for 2 min, 94 °C for 15 s, 60 °C for 15 s, 68 °C for 20 s. The number of PCR cycles was 34 followed by 68 °C for 1 min and 4 °C hold. Nonquantitive RT-PCR reactions were performed using cDNA from head, thorax, abdomen, and reproductive organs of male and female *An. coluzzii* mosquitoes. Testis and MAG semiquantitative RT-PCR experiments were conducted in *An. coluzzii* using 40S ribosomal protein S7 gene as a gel-loading control. We used equal volumes of cDNA for each experiment. PCR products were visualized using gel electrophoresis in 1% agarose gel. Each RT-PCR experiment was repeated three times to account for any random effects in the results. 

### 2.6. Selection Analyses for Molecular Evolution of Parental Genes and Retrogenes

To understand how selection has shaped the molecular evolution of parental genes and retrogenes, we searched for positive selection using two complementary types of models: the optimal branch specific model (OBSM, method 1) [43] and the branch-site model (BSM) [44]. The former was used to search for the optimal model of whole-gene molecular evolution across phylogenetic branches, and the latter was used to search for positive selections on our focal species *An. gambiae* by considering specific sites within a gene. For each retrogene of *An. gambiae*, its orthologous genes and its parental gene’s orthologous genes were incorporated. Finally, 39 groups of “one to one” orthologous genes with gene order conservation (GOC ≥ 25, confidence = 1) were retrieved from Ensembl Metazoa database (v53). The significant level of branch-site Model A for positive selection was based on a comparison of Model A (“model = 2” and “NSSites = 2”, fix_omega = 0) to Model A-null (model = 2, Nssites = 2, fix_omega = 1, omega = 1). For the OBSM, only perfectly nested models with the difference of only one degree of freedom were compared. All *p* values of the likelihood ratio test (LRT) were computed based on a nested model comparison with χ^2^ tests, where the testing statistic equaled two times the difference in log likelihood values, and the degrees of freedom was equal to the difference in the number of model parameters. 

## 3. Results

### 3.1. Retroduplications Occurred Predominantly from the X Chromosome to Autosomes after the Evolution of Differentiated Sex Chromosomes 

**Figure 2:**
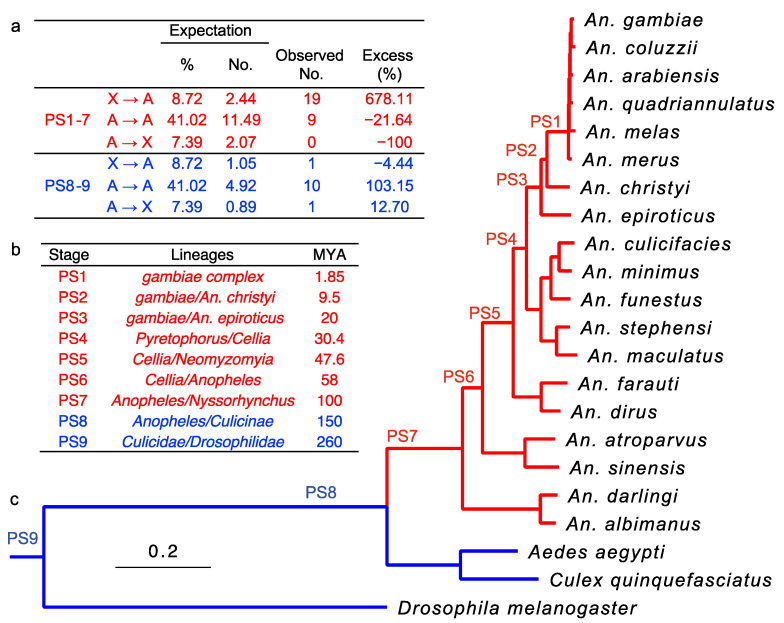
Retroduplication events distribution during mosquito evolution. (**a**) The expected frequencies, expected numbers, and the excess rates for three directions (X → A, A → X, A → A) organized by phylogenetic stages PS1–7 and PS8–9. A → X, from autosomes to X chromosome; X → A, from X chromosome to autosome; A → A, from autosome to a different autosome. In PS8–9, the directions of retroposition refer to the homolog of the differentiated X chromosome in PS1–7 lineages. The expected percentages and numbers were calculated based on the formula of Betrán et al. (2002) [8]. Note: for PS1–7, the permutation of 1,000,000 replicates of X^2^ test, *p* = 0.0025, whereas for PS8–9, *p* = 1. (**b**) Each phylogenetic stage is defined as a lineage with a previously estimated divergence time [34,35,36]. (**c**) Phylogenetic tree of evolutionary relationship denoting each defined phylogenetic stage [34]. The scale bar refers to genetic distances in nucleotide substitutions per site in all sites of single-copy genes.

We computationally identified a total of 40 interchromosomal retroduplication events that occurred during nine portions of the phylogenetic history of malaria mosquitoes (Table S3). We manually checked these pairs to ensure correct structural changes, specifically intron-loss signals, from parental genes to retrogenes (Supplementary file S1). Only one pair (AGAP000794-AGAP006891) was found to have a parental gene where the longest intron was shorter than 50 bp, indicating potential mis-assignment of the parental-derived relationship. We determined AGAP000794 to be the parental gene, based on the more conserved gene order synteny across species in AGAP000794 than in AGAP006891. Retroduplications were grouped by the direction of transposition (X to autosome, X → A; autosome to autosome, A → A; and autosome to X, A → X) (Figure 2a). Retroduplications were assigned relative ages based on presence-absence in particular genomes, i.e., phylogenetic stages (PSs) (Figure 2b). Using *An. gambiae* as the focal species, we determined the evolutionary age of retrogenes with a gene synteny-based method, where the most distant branch with gene presence and synteny support, i.e., conserved neighborhood for at least two genes was used to represent gene age [33].

We defined as young retroduplications those that occurred within the *Anopheles* genus and correspond to phylogenetic stages 1 through 7 (PS1–7) (Figure 2c), and include the species with heteromorphic sex chromosomes (X and Y chromosomes in males) [24]. The young stages of PS1–7 include 28 retroduplication events, including 67.86% X → A events (19), 32.14% A → A events (9), and no A → X events. Following the method of Betrán et al. (2002) [8], we estimated the expected frequencies, numbers, and excess retrotransposition rates (Figure 2a). It was important to consider dosage compensation when calculating retroposition events. Since the retroduplication process initiates from mRNA, more mRNA can increase the probability of retroposition events. Thus, considering dosage compensation of the X chromosome in *An. gambiae* [45] and the expected effective population size of autosomes (1) and X (0.75), we expected the percentage of X → A events to be only 8.72% versus 41.02% of A → A events. Subsequently, X → A events were estimated to be in excess by 678.11%: A → A and A → X by 21.64 and 100%, respectively. These results suggest a pattern of retroduplication “out-of-X” in young/heteromorphic sex chromosome *Anopheles* lineages. Due to the small sample size (Figure 2a), we performed 1,000,000 random permutations to test its significance; this revealed a significant difference between the observed and expected patterns of gene retroduplication between chromosome numbers of gene movement and those of expectation (*p* = 0.0025) [9]. These results are consistent with the trends observed previously for retrogenes in the presence of heteromorphic sex chromosomes in *Anopheles* [46]. 

In contrast, we defined older retroduplications by the more ancient stages 8 through 9 (PS8–9) that represent retroposition events in the lineages leading to the common ancestor of the *Anopheles* and the *Culicinae* subfamily and the mosquito and *Drosophila* lineages, which lack differentiated sex chromosomes [46]. In total, 12 interchromosomal retroduplications were identified (Figure 2a). Interestingly, the observed numbers of gene movements in the three directions before the sex chromosomes evolved, considering the chromosome homologies (see details above), were not significantly different from the null expectation of random events (permutation X^2^ test with 1,000,000, replicates, *p* = 1).

### 3.2. Most Retrogenes Originated after the Evolution of Sex Chromosomes Evolved Male-Biased Expression in Reproductive Organs whereas Older Retrogenes Acquired Female-Biased Expression

**Figure 3:**
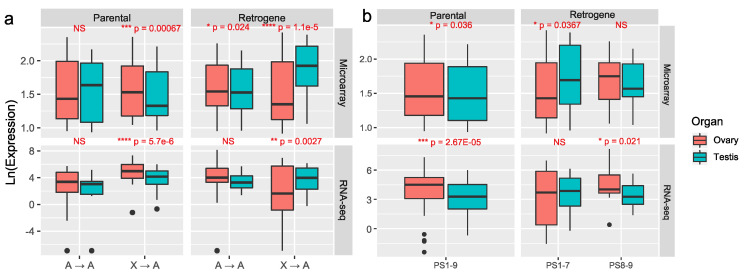
Microarray and RNA-seq expression values for *An. gambiae* retrogenes and their parental genes. (**a**) The expression comparison between testis and ovary for retrogenes and parental genes with movement directions of X → A and A → A; (**b**) The expression comparison between testis and ovary for parental genes and retrogenes with different ages. For the RNA-seq data, the ovaries sample also included common oviduct and the testis sample included accessory glands. The microarray and RNA-seq data are mean RMA ln of expression and the mean transcript abundance in transcripts per million (TPM + 10^−3^), respectively. The detailed expression values and standard errors were listed in Table S4. The *p* values were estimated based on the paired Wilcoxon signed rank test.

To explore why young retroduplications predominantly occurred from the X chromosome to autosomes, we analyzed expression patterns of parental genes and retrogenes in PS1–7. We hypothesized that, as observed in previous work (see Introduction), such retroduplications could have evolve male-biased expression. We tested whether the young X-to-autosome retroduplications in the *Cellia* subgenus of the genus *Anopheles* evolved male-biased expression in reproductive organs. For comparison, we analyzed expression patterns of old retroduplication gene pairs. Expression of retrogenes and parental genes was analyzed using the microarray data for *An. gambiae* testes and ovaries [39] and using the RNA-seq data for *An. gambiae* male reproductive organs (consisted of testes and MAGs) and female reproductive organs (consisted of ovaries and common oviduct) [40]. The normalized expression values for these experiments were taken from the VectorBase [41] (Table S4, Figure 3). Both microarray and RNA-seq data showed a similar pattern in sex-biased expression (Figure 3 and Table S4). 

We first focused only on sex-biased expression pattern for the two different movement directions (X → A or A → A) (Figure 3a). The parental genes demonstrated no sex bias for the A → A duplication pattern but showed significant female-biased expression in the X → A direction. The microarray data revealed that A → A retrogenes have significantly female-biased expression although the RNA-seq data did not. Both microarray and RNA-seq data supported male-biased expression for the X-derived (X → A) retrogenes. These results demonstrated that X → A retrogenes generally evolved to be male-biased whereas A → A retrogenes did not subsequently evolve male-biased expression.

We further analyzed expression differences between the sexes based on age groups (Figure 3b). The parental genes showed significantly higher expression in ovaries than in testes, which was also observed for ancient retrogenes (PS8–9). However, young retrogenes (PS1–7) demonstrated a different pattern where the testis, instead of ovary, was the higher expressed organ. In detail, for microarray data, sex-biased genes accounted for 62.96% (17/27 of retrogenes in young lineages PS1–7) because one of the 28 young PS1–7 retrogenes had no data, and 70% (7/10 of retrogenes at ancient stages PS8–9): although there were 12 ancient retrogenes in PS8–9, two genes had no data) (Table S4). Among the sex-biased genes (*p* < 0.05) [39], 64.71% of the young retrogenes (PS1–7, 11/17) were male-biased, versus only 14.29% of the older ones (PS8–9, 1/7). Thus, the young retrogenes (PS1–7) are 11 times more likely to have male-biased expression (odds ratio = 11; 95% CI; 1.06–114; *p* = 0.04). In addition, 66.67% (18/27) of the parental genes (PS1–7 and PS8–9) were female-biased, with no difference between the ancient (PS8–9, 6/10) and young (PS1–7, 10/17) stages (’Fisher’s exact test, *p* = 1) according to the microarray data. Unlike the pattern in the microarray data, RNA-seq revealed a higher fraction of female-biased (14/25) than male-biased (11/25) retrogenes at the young stage retropositions (PS1–7). However, over half of the male-biased genes (6/11) were in the youngest stages (PS1 to PS4), whereas the female-biased genes were predominately (12/14) in the older stages (PS5-PS7) according to RNA-seq. Thus, RNA-seq revealed, at a finer resolution than microarray, that the youngest retrogenes, which emerged at or after emergence of the *Cellia* subgenus (PS1–4), were predominately male biased. For genes with sex-biased expression in the RNA-seq data (*p* < 0.05), 72.73% (8/11) of ancient retrogenes at PS8–9 and 85.71% of parental genes (24/28) were female-biased, which is consistent with the expression pattern in the microarray data. 

When considering both age and direction of movement of sex-biased retrogenes, the A → A young (PS1–7) retrogenes were mostly female-based (80%, 4/5), whereas 83.33% (10/12) of the X → A young retrogenes were male-biased according to the microarray data (Table S4). Among the 12 X → A young sex-biased retrogenes, both PS1–4 and PS5–7 retrogenes were mostly male-biased (6/7 and 4/5, respectively) according to the microarray results. For the 17 RNA-seq-revealed sex-biased X → A young retrogenes, the younger stages (PS1–4) were more likely to be male-biased (75%, 6/8). This was different from the older stage (PS5–7) duplications which were mostly female-biased (66.67%, 6/9). Considering the heteromorphic nature of the sex chromosomes of the PS1 to PS7 species, this pattern suggests that either the expression of X → A retrogenes gradually evolved female bias or the male-biased genes were lost more often [47,48].

### 3.3. Autosomal Retrogenes with Increased Meiotic or Postmeiotic Expression Tend to Be Male Biased 

**Figure 4:**
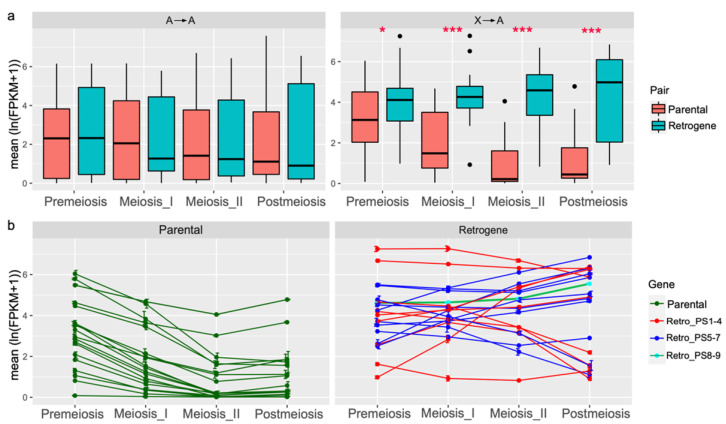
RNA-seq expression profiles of retroduplications pairs during spermatogenesis of *An. gambiae*. (**a**) Boxplot of mean ln(FPKM + 1) expression values for 40 retrogenes and 40 parental genes in four cell populations (A → A, left; X → A, right). *, *p* < 0.05; ***, *p* < 0.001. The *p* values were estimated based on a paired Wilcoxon signed rank test (one-tailed). (**b**) Mean ln(FPKM + 1) expression values (the natural logarithmic value) of 20 parental genes (left) and 20 retrogenes (right) of the X → A type in four cell populations.

To test whether meiotic sex chromosome inactivation (MSCI) may have influenced retroduplications in *Anopheles*, we analyzed RNA-seq data in four distinct cell lineages from *An. gambiae* male gonads: premeiotic cells, primary meiotic spermatocytes, secondary meiotic spermatocytes, and postmeiotic cells [28]. These RNA-seq expression data represent transcript presence, not transcription of a particular gene. This means that despite processes such as MSCI, which potentially silences some genes during meiosis, transcripts that were produced before the silencing occurred can still be present. Therefore, even genes on the X chromosome, which are expected to be silenced during meiosis, may have non-zero expression values for RNA-seq expression data. For the 40 retroduplications pairs within PS1-PS9, RNA-seq expression levels in premeiotic, meiosis I, meiosis II, and postmeiotic cells were compared between retrogenes and their parental genes (Table S5). For the A → A type gene duplication, no significant differences in expression values were found between the 20 retrogenes and their parental genes across the spermatogenesis stages (Figure 4a left). However, significant differences (*p* < 0.05) were found between the 20 X → A retrogenes and their parental genes across the four stages of spermatogenesis (Figure 4a right). For 19 X → A retrogenes at PS1–7, the median expression levels increased during spermatogenesis stages, whereas the X-chromosomal parental genes showed an apparently descending trend, consistent with the expectation of MSCI in species of PS1–7. 

All 20 parental genes of the X → A type had a consistent pattern of expression throughout spermatogenesis (Figure 4b left). The highest levels of transcription of these X-chromosomal genes were at the premeiotic stage with a gradual reduction in expression at the meiosis I and II cell populations. This pattern of expression is consistent with the observation of MSCI in *An. gambiae* [28]. Out of 20 X → A retrogenes, however, 13 (AGAP001767, AGAP005098, AGAP006891, AGAP010423, AGAP011684, AGAP005558, AGAP005981, AGAP007024, AGAP009572, AGAP007630, AGAP013199, AGAP028116, and AGAP005197) showed higher transcription levels in one of the stages after the premeiotic stage of spermatogenesis (Figure 4b right). Nine of these 13 retrogenes had male-biased expression in reproductive organs. Retrogenes AGAP005981, AGAP005197, and AGAP007024 had female-biased expression and AGAP009572 was male-biased by microarray but female-biased based on RNA-seq (Table S4). For the other seven X → A retrogenes that had decreased expression in meiosis I and II in comparison with the premeiotic stage, only AGAP004901 had male-biased expression in reproductive organs, whereas the remaining genes included one unbiased and five female-biased (Table S4). Among the seven retrogenes showing lower expression in one of the stages after the premeiotic stage, four genes emerged in PS1–4 and three in PS5 (Figure 4b right).

Thus, most of the young X → A retrogenes from PS1-RB7 (12/19, 63.16%) evolved increased meiotic or postmeiotic expression in spermatogenesis of *An. gambiae*, and 83.33% (10/12) of them had male-biased expression in reproductive organs. The remaining seven young autosomal retrogenes had increased premeiotic expression and five of them had female-biased expression in reproductive organs based on both RNA-seq and microarray data. This finding supports two contrasting tendencies, where male-biased expression tends to occur in genes with higher expression at the later stages of spermatogenesis, whereas female-biased expression is established for retrogenes with decayed expression at the later spermatogenesis stages (the Fisher exact test, *p* = 0.013). Considering the heteromorphic nature of the X chromosome in species within PS1–7, our analyses indicate that the MSCI, which necessitates the later-stage expression of spermatogenesis-related proteins, might be a strong force to drive the male-biased expression of retrogenes. 

### 3.4. Younger Retrogenes Have Nonuniform Expression Patterns across Body Parts and Sexes 

**Figure 5:**
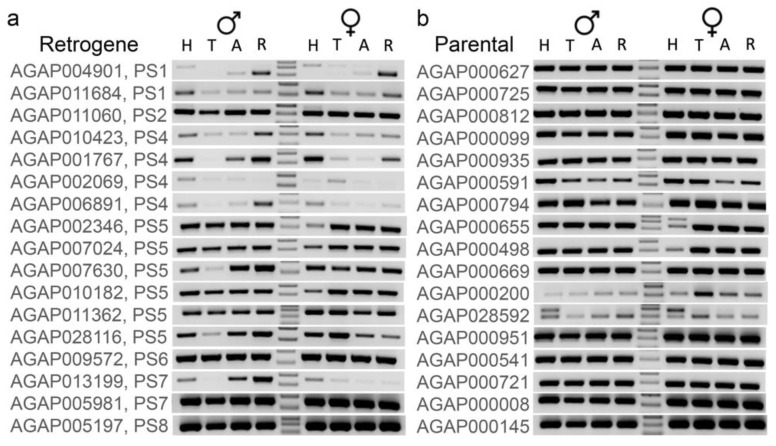
RT-PCR expression profiles of young retroduplication pairs from PS1-PS8 in head, thorax, abdomen, and reproductive organs of *An. coluzzii.* Gene names follow *An. gambiae* nomenclature. (**a**) Retrogenes. (**b**) Parental genes. H–head, T–thorax, A–abdomen, R–reproductive organs.

To test whether retrogenes have expression patterns in body parts and sexes different from those of parental genes, we performed RT-PCR experiments using *An. coluzzii.* *Anopheles coluzzii* and *An. gambiae* are the most closely related species in the *An. gambiae* complex and do not have postzygotic reproductive barriers [16,17]. Although these experiments were not quantitative, reactions were performed using the same amounts of template DNA from the same sources for different primer pairs. Therefore, expression patterns can be compared among genes. We specifically looked at the pattern of expression in heads, thoraxes, abdomens, and reproductive organs of males and females. For this experiment, we used 17 gene pairs with X → A retropositions in PS1-PS8. The RT-PCR experiments confirmed that these newly derived retrogenes are functional (Figure 5). A few retrogenes had no visible product or showed weak expression in some body parts. Of the seven youngest retrogenes (PS1–PS4), only one retrogene, AGAP011060, had uniform expression across all body parts in both males and females, although the female-biased expression was detected by microarray and RNA-seq data (Table S4). Some of the strongest RT-PCR bands for five retrogenes from PS1-PS4 (AGAP004901, AGAP011684, AGAP010423, AGAP001767, and AGAP006891) were observed in reproductive organs and heads of both males and females (Figure 5a). The microarray and RNA-seq studies of sex-biased expression showed that these retrogenes have male-biased expression in reproductive organs (Table S4). The remaining retrogene AGAP002069 showed very weak bands in both male and female reproductive organs and unbiased expression according to both microarray and RNA-seq. Unlike the youngest retrogenes, most of those from PS5-PS8 had uniform expression across body parts and sexes. Retrogenes AGAP007630, AGAP028116, and AGAP013199 had weak or non-visible RT-PCR product in one or more somatic body parts or female reproductive organs. In agreement with the microarray and RNA-seq sex-biased expression data (Table S4), these three retrogenes had male-biased expression in the reproductive organs, whereas the remaining older retrogenes had unbiased or female-biased expression (Table S4). All 17 cognate parental genes demonstrated the strong presence of RT-PCR products across all tested body parts and sexes (Figure 5b). 

**Figure 6:**
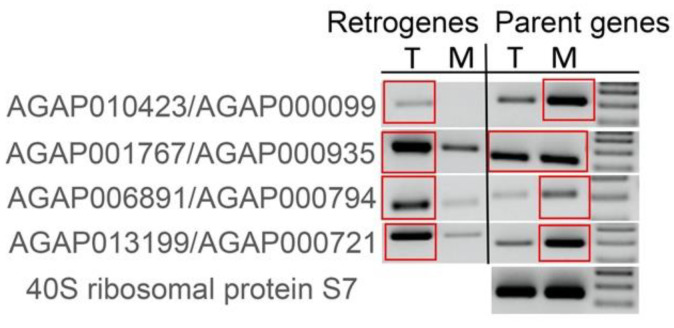
RT-PCR expression profiles of young retroduplication pairs in testes and MAGs of *An. coluzzii.* Red squares indicate strongest RT-PCR bands within each comparison. The 40S ribosomal protein S7 gene (AGAP010592) was used as a gel loading control. T–testis, M–MAG.

Male reproductive organs in the above RT-PCR experiments included testes and MAGs. To check if stronger RT-PCR bands in male reproductive organs were due to testis or MAG expression, we selected four retrogenes that showed the strongest RT-PCR product in male reproductive organs and looked at their expression separately in testes and MAGs. All four showed stronger RT-PCR bands in the testes than in MAGs. In comparison, the corresponding parental genes showed equal or stronger MAG expression (Figure 6). Thus, a subset of X → A retrogenes evolved testis-biased expression.

### 3.5. Mitochondrial Function Is a Distinctive Feature of the Young Retrogenes

To gain insights into possible functions of retrogenes, we obtained their functional annotations from VectorBase (Table S6) and from the *Kyoto Encyclopedia of Genes and Genomes* (KEGG) [49] (Table S7). We specifically looked at annotations of the 19 young X → A retrogenes from PS1-PS7. Of these, nine genes were mapped into the KEGG database with clusterProfiler v4.0, creator: Guangchuang Yu; source location: Shanghai, China [50]. Interestingly, the enrichment analysis suggested a significant over-representation of the pathway “oxidative phosphorylation,” which takes place in mitochondria (AGAP006891/AGAP005098/AGAP013199, *p* = 0.0039, adjusted *p* = 0.031) (Table S8). Considering the limited power of gene ontology enrichment due to the small number of genes mapped in clusterProfiler, we manually reviewed the functions of the remaining annotated X → A PS1-PS7 retrogenes (Table S7). Several of them have potential functions in mitochondria based on KEGG annotation. For example, AGAP004901 is involved in ATP binding and protein kinase activity. AGAP005558 and AGAP001767 encode for proteins that are part of the mitochondrial processing peptidase complex involved in metalloendopeptidase activity. Functional annotations of these retrogenes support the biological enrichment of mitochondria-related processes.

Other retrogenes have functions probably unrelated to mitochondria. For example, AGAP011684 encodes a protein involved in protein peptidyl–prolyl isomerization. A product of AGAP002069 possesses prenyltransferase activity and is involved in isoprenoid biosynthetic process. AGAP011060 has protein serine/threonine kinase activator activity and participates in intracellular signal transduction. AGAP010423 has a function in regulation of transcription by RNA polymerase II and in the receptor-signaling pathway via JAK-STAT. This retrogene is known as STAT1 [34] or STAT-B [51] and was found to be activated in response to bacterial challenge [52]. The JAK-STAT pathway may play a role in killing *Plasmodium falciparum* parasites at the oocyst stage in *An. gambiae* [53]. Two retrogenes, AGAP002346 and AGAP009572, encode proteins of cytosolic small ribosomal subunit with function in translation. AGAP010182 has a protein-binding function of the Cul4-RING E3 ubiquitin ligase complex. AGAP007024 is located in the nuclear pore central transport channel and plays a role in protein import into nucleus. AGAP005981 is involved in Hsp70 protein binding. AGAP001701 is involved in ubiquitin-like protein ligase binding and protein sumoylation; AGAP007630 is an integral membrane component; AGAP028116 has a protein-binding function; and AGAP011362 plays a role in chromatin organization inside the nucleus.

### 3.6. Most of the An. gambiae Retrogenes Show Signatures of Purifying Selection

We searched the optimal branch model of molecular evolution for 39 groups of retrogenes and their parental genes based on OBSM [43] (Table S9). The gene groups comprised the “one-to-one” orthologous genes of *An. gambiae* retrogenes and parental genes across the phylogeny (Figure 2c). Our analysis revealed that 20 groups had significant signals of positive selection (dN/dS > 1, *p* < 0.05) at the external/extant or internal/ancestral branches and the rest 19 groups were under purifying selection at all branches (dN/dS < 1, *p* < 0.05). Among the 20 positively selected gene groups, 60% (12/20) comprised either retrogenes or parental genes exclusively. Of these exclusive genes, 66.7% (8/12) were retrogenes positively selected at one or more extant or ancestral branches. Furthermore, 62.5% (5/8) of the retrogenes were found to be positively selected at ancestral branches while negatively selected at the extant/external branches. 

For our focal species *An. gambiae*, we compared dN/dS ratios between retrogenes and their parental genes based on the optimal model of OBSM. We found no significant deviation of dN/dS ratios between retrogenes and parental genes for most of retrogenes (36/39) of *An. gambiae*. All these genes were under the functional constraints of purifying selection based on dN/dS ratios (dN/dS < 1), thus supporting these retrogenes as functional genes rather than pseudogenes. Two retrogenes at PS1 (AGAP004901 and AGAP011684) and one retrogene at PS7 (AGAP005981) had higher dN/dS ratios than their parental genes at the *An. gambiae* branch, suggesting that they were under relaxed-purifying or positive selection to enable the functional divergence in *An. gambiae*. Interestingly, a positive selection was found for AGAP011684 (dN/dS = 1.283). According to the VectorBase [41] annotation, it was an aryl hydrocarbon receptor-interacting protein, a binding partner of transcription factor IRF7, which plays antiviral roles [54]. Consistent with a previous study [51], we found signals of positive selection based on branch-site model analyses in AGAP010423 (STAT-B), which is involved in resistance to bacteria and *Plasmodium* parasites.

## 4. Discussion

The first important observation of this study is that the retroduplication events in PS1-PS7, i.e., after the evolution of differentiated sex chromosomes, occurred predominantly from the X chromosome to autosomes. This direction of retropositions was previously observed in whole genome studies of retrogenes in fruit flies and mammals and was termed the “out of the X” pattern [8,9,10]. In contrast, no chromosome was favored for old retroduplications (PS8-PS9), i.e., lineages before sex chromosomes evolved. The observed pattern of retropositions allowed us to explore factors that may have influenced the evolution of retrogenes after the evolution of differentiated sex chromosomes. We specifically looked at the role of sexual antagonism [14,15,16,17] and meiotic sex chromosome inactivation (MSCI) [19,20] in favoring retropositions from the X chromosome to autosomes in those lineages.

We found that X → A retrogenes from the youngest branches (PS1 to PS4) evolved male-biased expression; however, X → A retrogenes from the relatively older stages (PS5–7) were mostly female-biased based on the RNA-seq data despite the presence of dimorphic sex chromosomes (Table S4). This could be explained by the gradual evolution of broader expression as retrogenes age or by male-biased genes being lost more often [47,48]. Previous reports uncovered a global pattern of female-biased gene expression in *An. gambiae* [40,55]. Our analysis revealed that this pattern is true only for relatively older X → A retrogenes, not those that evolved in the PS1–4 stages, and that the proportion of female-biased genes gradually increased with the age of the retrogene. The cognate parental genes as well as the old retrogenes and their parental genes had predominantly female-biased expression in the reproductive organs and male-biased expression of the youngest retrogenes. Previous studies of retrogene duplication in *Drosophila* and mammalian genomes also described the specific transcription of autosomal retrogenes in the male germline and the broad transcription of X-chromosome parental genes [8,9,10]. It was suggested that autosomal retrocopies may compensate for the repression of X-linked parental genes in the male germline due to MSCI [8,9]. MSCI was first described in mammals as a packaging mechanism for unpaired chromatin as meiosis proceeds [19,56]. Our analysis showed that, as expected, if the MSCI occurred for parent genes in *An. gambiae* [28], all of the parent genes that were duplicated in events during the phylogenetic stages PS1-PS7 had their highest levels of transcription at the premeiotic stage and lower expression in the meiosis I and II cell populations. In contrast, of 20 such X → A retrogenes, 13 showed the highest transcription in the meiosis I or postmeiotic stages, suggesting that their expression changed after they moved from the X. Nine of them had male-biased expression in the reproductive organs, making it plausible that MSCI acted in these species, which had evolved heteromorphic sex chromosomes, and could have led to selection for retroduplication from the X chromosome to an autosomal location, allowing expression during male meiosis. In addition, the postmeiotic expression could also be interpreted under the existence of haploid selection [57] as has also been proposed for new *Drosophila* genes [58,59].

In contrast, there was no male-bias for older retropositions (PS8-PS9) in species with homomorphic sex chromosomes [24,38] (e.g., *Aedes* and *Culex*). Strong male–female differentiation in the 63 Mbp region of chromosome 1 was not accompanied by significantly differentiated gene expression between males and females in the same region [60], indicating that these are still typical homomorphic sex chromosomes. Thus, we concluded that homomorphic sex chromosomes in PS8-PS9 likely behaved similar to autosomes that did not have sex-related sexual antagonistic selection or MSCI. This reasoning explains the observed excess of X → A retroduplications in PS1-PS7 but not in PS8-PS9. 

Young retrogenes had a variety of annotated molecular functions, some of which are related to mitochondrial processes (Tables S6 and S7). Indeed, the enrichment analysis resulted in the significant over-representation of the “oxidative phosphorylation,” pathways, which takes place in mitochondria (Table S8). It is known that mitochondria-related events are involved in multiple stages of reproductive function [61]. It is also known that the mitochondria of spermatozoa are generally significantly different from somatic mitochondria, at least in humans [62]. Additionally, a recent study suggested that *Drosophila* males might use different mitochondria in their germline. The study demonstrated that retrogene COX4L has energy-related functions, testis-biased expression, and is essential for male fertility, whereas its parental gene COX4 does not have function in *Drosophila* testes [63]. The observed enrichment of mitochondrial functions in retrogenes suggests that there are likely additional strong selective pressures at work in the male germline. The testis-biased expression of genes with a function in mitochondria may be reflective of a resolution of an intralocus sexually antagonistic conflict [16,17]. The faster evolution of these genes right after duplication, including some with dN/dS values higher than 1, supports that these genes might be gaining a new function that might not be completely consistent with MSCI but rather with the resolution of sexual antagonism [16].

Males usually do not pass on mitochondria to their offspring and are under strong competition with other males to fertilize eggs. These male features create a selective pressure that favors mitochondria with high-energy production despite the associated risk of increased generation of reactive oxygen species and mutations [17,64,65]. The detrimental effects of the high energy-producing genes expressed in the soma or ovary outweigh their benefits: they cause faster aging in the soma [66], and offspring inherit damaged mitochondria via the ovaries [67]. This situation would generate an intralocus sexually antagonistic conflict that might be resolved through the fixation and maintenance of testis-specific retrogenes. The fact that some of the young male-biased retrogenes in *Anopheles* have molecular functions in mitochondria suggests that the selective pressures to preserve these retrocopies have been very specific. Similar retrogene functions have been observed in *Drosophila* and in mammals [1,65,68,69,70,71]. Studies in *Drosophila* have demonstrated that past sexually antagonistic conflict related to mitochondrial energy function can be resolved by gene duplication [65,72]. In *Anopheles*, such conflicts might have been resolved via *the* retroduplication of genes with mitochondrial functions from the X chromosome to autosomes.

Among other young X → A retrogenes, AGAP010423 encodes a STAT protein involved in the highly conserved transcription regulation pathway known as JAK/STAT [73]. This gene was found to play a role in mosquito innate immunity [52]. In the human JAK/STAT pathway, multiple STAT proteins have been observed in sperm components suggesting a role in sperm function [74]. In *D. melanogaster,* the JAK/STAT pathway contributes to maintaining adult somatic stem cells in adult testes [75]. Molecular analysis of AGAP010423 and its parental copy showed strong evidence of adaptive evolution that is consistent with neofunctionalization after retroduplication [51]. Based on the evidence of JAK/STAT involvement in multiple aspects of male fertility, it is likely that AGAP010423 was positively selected for a unique role in *Anopheles* spermatogenesis. 

**Figure 7:**
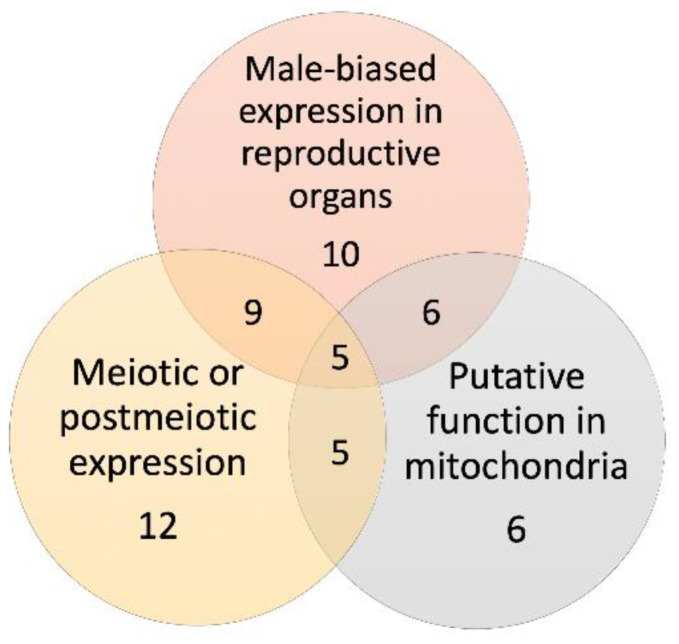
A Venn diagram showing overlaps in the number of young X → A retrogenes among three groups of tested and predicted functional characteristics.

Thus, our study identified three common characteristics among 19 young (PS1–7) X → A retrogenes in malaria mosquitoes: male-biased expression in reproductive organs, the highest expression after the premeiotic stage, and molecular functions in mitochondria (Table S10). We asked to what degree these characteristics overlap among genes. We found that 9 of 10 retrogenes with male-biased expression in reproductive organs also had the highest expression at meiotic or postmeiotic stages. Furthermore, all six retrogenes with molecular functions in mitochondria had male-biased expression in reproductive organs and the highest expression in meiotic or postmeiotic cells (Figure 7). Thus, a combination of sexual antagonism and MSCI-influenced evolution of the young X → A retrogenes is likely.

Our RT-PCR data indicated that the X → A retrogenes, especially the youngest (PS1–4) ones, evolved non-uniform expression patterns across body parts in both males and females (Figure 5). This is in contrast with the more ubiquitous pattern of expression of the older X → A retrogenes and cognate parental genes. This pattern of expression was consistent with observations of many retrogenes, particularly young ones, tending to have lower transcription levels in most tissues and narrow expression breadth [76]. A significant fraction of young retrogenes in *Anopheles* had the strongest expression primarily in the male germline (Table S4). Our data support the “out of the testis” hypothesis implying that young retrogenes are more often testis-specific compared to old ones [76,77,78]. This hypothesis has two alternative, but not mutually exclusive, potential explanations. First, high transcription levels in the male germline could be facilitated by a permissive chromatin state during the transition from standard histones to testis-specific histone variants [79]. As a result, young retrogenes “take advantage” of this chromatin state shift in the testes for their expression, which they do not have in other tissues. Expression levels of retrogenes tend to increase in other tissues as they adapt to new chromatin environments with age [1]. Second, the age effect on expression can also be a consequence of a high rate of gains and losses of testis-specific genes as opposed to broadly expressed genes. A high turnover of testis-specific genes can be a consequence of selective pressure caused by male–male competition, male–female antagonism, and host defense against infections or selfish genetic elements [47]. Interestingly, the expected high expression of multiple young retrogenes in male reproductive organs, specifically in the testes (Figure 6), was complemented by high expression of some retrogenes in the heads of both sexes (Figure 5). The high head expression could suggest new functions of retrogenes in some aspects of mating behavior. Our RT-PCR experiments showed that young retrogenes have stronger testis rather than MAG expression, whereas the cognate X-linked parental genes have an unbiased or MAG-biased pattern of expression (Figure 6). MAG proteins are an essential component of seminal fluid deposited into the *An. gambiae* female reproductive tract during copulation and they play important roles in inducing female post-mating responses, including oviposition and monogamy [80,81,82,83]. An over-representation of MAG-biased genes on the X chromosome [26] suggests that components of male reproductive biology in malaria mosquitoes can be antagonistic. Future investigations of retrogenes in *Anopheles* may reveal new gene functions important for mosquito reproduction that can be exploitable in genetic approaches to vector control. 

## Data Availability

Not applicable.

## Supplementary Materials

The following supporting information can be downloaded at: https://www.mdpi.com/article/10.3390/genes13060968/s1, The supplementary file S1: The retrogene-parental gene alignments and statistical notes; Table S1: Species and gene annotation datasets used for identification of retrogenes and parent genes; Table S2: Sequences of forward (F) and reverse (R) primer pairs for RT-PCR of retrogenes and parental genes of *An. coluzzii*; Table S3: A list of retrogenes and parental genes with their phylogenetic stages, chromosomal location, and % identity; Table S4: Expression of retrogenes and parental genes in reproductive organs of *An. gambiae*; Table S5: RNA-seq expression profiles of retroduplications pairs in spermatogenesis of *An. gambiae*; Table S6: VectorBase community functional annotations of young X - > A *An. gambiae* retrogenes and parental copies; Table S7: KEGG functional annotations of young X → A retrogenes of *An. gambiae*; Table S8: KEGG enrichment result for *An. gambiae* retrogenes functions with clusterProfiler v4.0; Table S9: Selection analyses for molecular evolution of parental genes and retrogenes; Table S10: Co-occurrence of common characteristics of the young X → A *An. gambiae* retrogenes.
