# Supplementary material for: Retrogene Duplication and Expression Patterns Shaped by the Evolution of Sex Chromosomes in Malaria Mosquitoes
Source: Genes (Basel). 2022 May 28;13(6):968. doi: 10.3390/genes13060968 (PMC9222922; doi:10.3390/genes13060968)
Supplement: Supplementary file 1 [file genes-13-00968-s001.zip › Supplementary file 1.pdf]

The supplementary file 1. The retrogene-parental gene alignments and statistical notes.

Content

The retrogene-parental gene alignment 1

The statistical note 14

The retrogene-parental gene alignment

1. AGAP010423-PA BR13L AGAP000099-PC X 46.543 0

```
  1 ETTKQLSVSFRNMQLKKIKRAEKKGTECVMDEKFALLFQSSFAVGHGDLVFSVWTISLPVVVIVHGNQEPQSWATITWDNAFADINRIPFQVPDKVIWNQ
  1          | || |||| |   |    | ||||||||||||      |   ||| ||| ||||| |||   | || |||  |   |  | ||    || 
  1 QLSNKFLANFSNMRLKKINRGNRKLNKLVVDEKFALLFQSSFTLEQEELTVTVWTLSLPAVVIVHVNQEQLAWTTIIWDNLCAKADRKLFEVPNLIPWNR

101 LAEALNMKFRASTGRSLTAENMHFLCEKAFKTNLPFPVPNDLTIMWSQFCKEPIPDRSFTFWDWFYAAMKVTREHLRGPWMDGSIIGFIHKSKAEDYLLK
101 | ||  | | |  || || |||     ||    | | | ||  |   ||||   |    ||| | ||| |  | ||   | |  |||||||| || || |
101 LVEAISMTFSARVGRGLTDENMQYMYRKAYRDKLSFSVSNDQMISFAQFCKDTTPECNYTFWEWLYAALKIIRDHLQVLWVDNTIIGFIHKSTAEKYLAK

201 CPRGTFLLRFSDSELGGITIAWVNEGNDGQPQILHIQPFTAKDFSTRSLSDRIRDFDDLFYLYPNKPKHEAFDRYTTPA
201 |  ||||||| || |||| |||| | |||| | ||||||||||   |||  || |   | ||||  || ||| ||| ||
201 CVPGTFLLRFTDSVLGGISIAWVHESNDGQRQVLHIQPFTAKDLVVRSLANRICDLGELTYLYPTIPKQEAFGRYTAPA
```

2. AGAP011684-PA BR13L AGAP000725-PA X 78.571 5.06E-180

```
                         10        20        30        40        50        60        70        80
AGAP00           MDSTLQEPKIVKNIVHAGTKVVPFRDGTKVKFHYQTRKCDEARTLIDDSRAHGQKPMELVLGKKFKLEVWESIVQQMALHEVARFRCDR
                 :: ::.. .:::..::.::: .::: :::: :::.:::::  :::::.::.: ::::.:::::.::::::::::::::.:::::::::.
AGAP01 MVPPLYRLEERMDYTLRDAQIVKTVVHVGTKFIPFRVGTKVTFHYETRKCDAERTLIDESRTH-QKPMQLVLGKQFKLEVWESIVQQMGLHEVARFRCDK
               10        20        30        40        50        60        70        80        90

      90       100       110       120       130       140       150       160       170       180
AGAP00 SLVQQYPFVSKTIRDAQKPRE--ERKHCCGMTVQNEGIGYRDLDELFTHPQDLEFTIEILSIESPDEYEKESWQLSDDEKRALVGRLREQGNAAYRANDL
       ::::::::::::::::::  .  .::::::.: ::.: ::::::: :::::::::::::::::.:::: ::.:::::::::.::.:::::::.:::.:.:
AGAP01 SLVQQYPFVSKTIRDAQKQPQGGKRKHCCGLTFQNDGTGYRDLDELFIHPQDLEFTIEILSIETPDEYGKEAWQLSDDEKRTLVSRLREQGNVAYRTNNL
     100       110       120       130       140       150       160       170       180       190

       190       200       210       220       230       240       250       260       270       280
AGAP00 TAARDAYSYATGIIEQLMLKEKPDEPEWIELAQMKVPLLLNYSQCKLLERDYYAAIEHCTEALKYDPHCVKALFRRGKAHAGAWNFDRARADFERAAELD
       :::::::::: ::::::::::::: ::::::::::::::::.:::::::.::: .::::::.:::::.::::::::::.. :...:: ::.:.::::
AGAP01 TAARDAYSYAIGIIEQLMLKEKPHEPEWIELAQMKVPLLLNFSQCKLLEQDYYPVIEHCTEVLKYDPQCVKGLFRRGKAHAAVCNYEQARNDFQRVAELD
     200       210       220       230       240       250       260       270       280       290

       290       300       310       320
AGAP00 SGLQTAVGKELAKLQEQQRLRDVEDRLKYQKLF
       ..: ::. ::::::..::::::::.::::: :.:
AGAP01 TSLGTAMRKELAKLKDQQRLRDVDDRLKYCKFF
     300       310       320       330
```

3. AGAP011060-PA BR2 3L AGAP000812-PA X 93.675 0

```
>>>AGAP000812-RA, 336 aa vs sgl library

>>AGAP011060-RA;3L;1:1017:15805306:15806325                          (339 aa)
 s-w opt: 2006  Z-score: 1947.8  bits: 368.8 E(1): 1.1e-106
Smith-Waterman score: 2006; 93.7% identity (97.6% similar) in 332 aa overlap (1-332:1-332)

               10        20        30        40        50        60        70        80        90       100
AGAP00 MPLFGKSQKSPQELVKALKEAVNSLERGDKKAEKAQEDVSKNLVLIKNMLYGTSDAEPQTEIVVSQLAHELYSTNLLLLLIQNLNRIDFEGKKDVAQIFN
       ::::::::::::::::.::::::.:::::::::::::::::::: ::::: ::.:::::::::.::::::::::.:::::::::::::::::::::.:::
AGAP01 MPLFGKSQKSPQELVKSLKEAVNALERGDKKAEKAQEDVSKNLVSIKNMLCGTADAEPQTEIVISQLAHELYSTDLLLLLIQNLNRIDFEGKKDVAHIFN
               10        20        30        40        50        60        70        80        90       100

              110       120       130       140       150       160       170       180       190       200
AGAP00 NVLRRQIGTRSPTVEYICTKPEILFTLMAGYEHQEIALNCGTMLRECARYEALAKIMLHSDEFFNFFRYVEVSTFDIASDAFSTFKELLTRHKILCAEFL
       ::.::::::: :::::::::::::::::::::::::::::: :::::::.::::::::::.::::::::::::::::::::::::::::::::.: ::::
AGAP01 NVIRRQIGTRLPTVEYICTKPEILFTLMAGYEHQEIALNCGIMLRECARHEALAKIMLHSEEFFNFFRYVEVSTFDIASDAFSTFKELLTRHKLLSAEFL
              110       120       130       140       150       160       170       180       190       200

              210       220       230       240       250       260       270       280       290       300
AGAP00 EQNYDKVFNHYEHLLNSENYVTRRQSLKLLGELLLDRHNFTVMTKYISNPDNLKLMMNMLKEKSRNIQFEAFHVFKVFVANPNKPKPILDILLRNQEKLV
       ::::::::..:: ::::::::::::::::::::::::::::::::::::::::::::::::: :::::::::::::::::::::::::.:::::::::::
AGAP01 EQNYDKVFSRYEALLNSENYVTRRQSLKLLGELLLDRHNFTVMTKYISNPDNLKLMMNMLKETSRNIQFEAFHVFKVFVANPNKPKPIMDILLRNQEKLV
              210       220       230       240       250       260       270       280       290       300

              310       320       330
AGAP00 DFLTRFHTDRSEDEQFNDEKAYLIKQIKELKPAPDQ
       :::::::::::::::::::::::: :::::::
AGAP01 DFLTRFHTDRSEDEQFNDEKAYLIMQIKELKPQTQEAQQ
              310       320       330
```

4. AGAP001767-PA BR3 2R AGAP000935-PA X 78.692 0

```
        10        20        30        40        50        60        70        80        90       100
MASLLKFSAGLRAYSRSHQLLFRRTKVSNAAEFRAALVNVPPTEVTTLDSGLRVASEDSGSQTATVGLWIDAGSRYENDSNNGVAHFLEHMAFKGTAKRS
: :::...  .:. :...: .. ::::..::.::.::.:.: :.:: ::.::::::::::..:::::.::.:::: ::.:::::::::::::::::::::
MFSLLRLANRVRVCSHTQQRMLGRTKVTDAAKFRTALANLPSTQVTQLDNGLRVASEDSGAETATVGVWINAGSRCENSSNNGVAHFLEHMAFKGTAKRS
        10        20        30        40        50        60        70        80        90       100

       110       120       130       140       150       160       170       180       190       200
QTDLELEVENMGAHLNAYTSREQTVFYAKCLSKDVPKAVEILSDIIQHSKLGEAEIERERGVILREMQEVESNLQEVVFDHLHATAYQGTPLGNTILGPT
:..:::::::.:::::::::::::::::::::::: :::::::::.:.  ::: :: ::: ::::::::.::::.:::::::::::.::: ::..::::.
QANLELEVENLGAHLNAYTSREQTVFYAKCLSKDVAKAVEILSDIVQNPTLGEEEIVRERDVILREMQEIESNLKEVVFDHLHATAFQGTALGKSILGPS
       110       120       130       140       150       160       170       180       190       200

       210       220       230       240       250       260       270       280       290       300
KNIQSIGKSDLQQYIDAHYKAPRIVLAAAGGVRHGDLVRLAEQALGKVSSSVDGKAAALAPCRFTGSEVRVRDDSLPLAHVAIAVEGCGWTDQDNVPLMV
::::::::..:..:::..:::::::::::::: : .::.::.: .:...: ::.:  ::  ::::::::::::::::::::.::::.:::::.:.:::::
KNIQSIGKTELKHYIDTQYKAPRIVLAAAGGVDHKELVQLAKQNFGEMNSIVDAKKDALDACRFTGSEVRVRDDSLPLAHVVIAVESCGWTDEDHVPLMV
       210       220       230       240       250       260       270       280       290       300

       310       320       330       340       350       360       370       380       390       400
ANTLIGAWDRSQGGGANNASKLAMASATDGLCHSFQSFNTCYKDTGLWGIYFVCDPLKCEDMLFNVQNEWMRLCTMVTEGEVERAKNLLKTNMLLQLDGT
:...::::::.:.:..:.:::::.:::.::.::::::::.::.:::::::::::::: :::::::::::::::::.:::::.::::::::::::::.:::
ATSFIGAWDRAQSGSVNHASKLAVASAVDGMCHSFQSFNVCYRDTGLWGIYFVCDPLTCEDMLFNVQNEWMRLCTIVTEGEIERAKNLLKTNMLLHLDGT
       310       320       330       340       350       360       370       380       390       400

       410       420       430       440       450       460       470
TPICEDIGRQMLCYNRRIPLHELEQRIDSVTAQNVRDVAMKYIFDRCPAVAAVGPVENLPDYVRIRSSMYWTRL
::::::::::.::::::::.::.::::::::: .::.:::::::::::::::::::::::::.::::::.::::
TPICEDIGRQLLCYNRRIPVHEMEQRIDSVTAAKVREVAMKYIFDRCPAVAAVGPVENLPDYMRIRSSMHWTRL
       410       420       430       440       450       460       470
```

5. AGAP002069-PA BR3 2R AGAP000591-PA X 70.19 0

```
        40         50         60         70                  80         90        100        110        120
GQQQQQRDLSLPASGPVVNVGGDGGGGGGHRAALLSAGSGGTPLVAH---------SVSGPIKRSFYTGRGNFIQPAAALAPARAGVRAPVGTGALFEL
                         MIPLHNQALLLSCRIAGT-LLCHINPAGWRTPTIATVARSAIHSTASKRFLPSQRVKPIAA---QPIGSA----L
                                 10         20         30         40         50         60

       130        140        150        160        170        180        190        200        210        220
NQKCSATANLHPCMRGYSSVHTQQPAGPIREYNIDPYILLEDELKYIFEDIRQEISRATNHQELNKIAVYYFDGQGKAFRPMVAILMAKALNYHMHNENS
QKSCSVPQHL--CTRQHETV-------------DPYVLLEGELRHIYQDIREEIELATNHPELGRIATYYFDGQGKALRPMITILMAKAL-----HSDR
 70         80                     90        100        110        120        130        140

       230        240        250        260        270        280        290        300        310        320
DVMNAQRQIAMISEMIHTASLVHDDVIDQSFARRGKPSVNVLWNHKKVTQAGDYILAVASMLLARLKHDEVTHILSQVLTDLVQGEFMQLGSKETENERF
PITKGQRQIAMISEMIHTASLVHDDVIDQSFARRGKPSVNVLWNQQQVAQAGDYILAVVSMMLARLQHNDVTLILSEILADLVQGELMQLDTKETENERF
 150        160        170        180        190        200        210        220        230        240

       330        340        350        360        370        380        390        400        410        420
AHYFTKTYRKTASLIANSLKAVAVLSGADEQMAELSFQYGRNLGLAFQFVDDLLDFVSSSEAMGKPAAADLKLGLATAPVLFACEKFPELNPMILRRFRE
AHYFARSYRKTASLIANSLKAVAVLTGVGERTAEISFQYGRNLGLAFQLVDDLLDFVSSAEAMGKPAAVDLKLGLATAPVLFACEQFPELNAMIVRRFRG
 250        260        270        280        290        300        310        320        330        340

       430        440        450        460        470        480
PGDVERAYELVHQSQGLEQTRFLARKHCIEALRLASQISESPYQKGLIVVGDFVLNRMK
EGDTERAYELVHRSEGLEQTRFLARKHCAEARRLASQFDQSPYQHALVVVCDFVLNRMK
 350        360        370        380        390        400
```

6. AGAP004901-PA BR3 2L AGAP000627-PA X 56.55 7.44E-126

```
               10         20         30         40         50         60         70         80         90        100
AGAP00 MNDFIVAGKYRIIRKIGSGSFGDIYLGINITNGEEVALKVESILARHPQLTYEYKLYKVLTGGVGIPHIRYFGQERSYHVLVMDLLGPSLEDLFNFCSRH

              110        120        130        140        150        160        170        180        190        200
AGAP00 FTIKTVLMLVDQMIGRLEYLHMKNFIHRDIKPDNFLMGIGRHCNKLFLIDFGLAKKYRDFRSRIHISYREDKNLTGTARYASINAHLGIEQSRRDDMESL
AGAP00                                                                                           SRRDDLESL

              210        220        230        240        250        260        270        280        290
AGAP00 GYVMMYFNRGSLPWQGLKATNKKQKYEKISEKKMSTPIEVLCKGFPAEFAMYLNYCRSLRFEEGPDYMYLRQLFRILFRTLNHQYDYTFDWTIMKQ----
AGAP00 GYVLMYFNLGTLPWQGLKAANKRQKYERISEKKLSTPVEELCKGYPREFSLYLAYCRHMDFIQRPDYCYLRKLFRTLFHRQGFVYDYVFDWNMLKFGRPN
     10         20         30         40         50         60         70         80         90        100

              300        310        320        330
AGAP00 ----RAMQQGSNNPPAVTSGENKREERRDKDKLSSDTDE
AGAP00 LNAFTSMKRGQSRTPSAT
     110        120
```

7. AGAP006891-PA BR3 2L AGAP000794-PA X 89.172 0

```
       10         20         30         40         50         60         70         80         90
MAFSVLNTVAKRTAANVYVTGGGLLKNVAALCN-GQQPARSAGKWFPDEQFIDQFKGPVMYPDEVTSRWKLPPWNSKIAPVEKTVRN-SLNFGPQHPAAH
MAFCVLNAAAKRTPTTVYVVGRGLLQNVTAPYRLHQQPARAAGKWFPDEQFIEQFKGPVMYPDHVTARWKLPPWNSKIAPVEKQVRNLKINFGPQHPAAH
       10         20         30         40         50         60         70         80         90        100

.00        110        120        130        140        150        160        170        180        190
GVLRLVLELDGETVMRADPHIGLLHRGT-KLIEYKTYTQALPYFDRLDYVSMMCNEQCYSLAVEKLLNIDIPLRAKYIRVLFAEITRILNHIMAVGTHAL
GVLRLVLELDGETVMRADPHIGLLHRGTEKLIEYKTYVQALPYFDRLDYVSMMCNEQCYSLAVEKLLNIEIPPRAKYIRVLFAEITRILNHIMAIGTHAL
      110        120        130        140        150        160        170        180        190        200

200        210        220        230        240        250        260        270        280        290
DVGALTPFFWLFEEREKMMEFYERVSGARMHAAYIRPGGVSQDLPLGLLDDIYEFASKFGERLDEVEDVLTTNRIWVQRTVDIGVVSAEDALNYGFSGVM
DVGALTPFFWLFEEREKMMEFYERVSGARMHAAYVRPGGVAQDIPLGLLDDIYDFAVRFGERLDEVEDVLTSNRIWVQRTVNIGVVTAEDALNYGFSGVM
      210        220        230        240        250        260        270        280        290        300

300        310        320        330        340        350        360        370        380        390
LRGSGIKWDLRKSQPYDAYDLVEFDVPIGTKGDCYDRYLCRVEEMRQSLRIIDQCLNQMPAGEIKTDDAKLTPPSRGEMKHSMEALIHHFKLFTQGYQVP
LRGSGIKWDLRKVQPYDAYDQMEFDVPIGTKGDCYDRYLCRIEEMRQSLRIIDQCLNRMPAGEIKTDDGKISPPSRTEMKQSMEALIHHFKLFTQGYQVP
      310        320        330        340        350        360        370        380        390        400

400        410        420        430        440        450        460
PGATYTAVEAPKGEFGLYVVSDGSSRPYRCKIKAPGFAHLAALDKIGRHHMLADVVAIIGTLDVVFGEIDR
PGSTYTAVEAPKGEFGVYLVSDGSSQPYRCKIKAPGFAHLAALDKIGRHHMLADVVAIIGTLDVVFGEIDR
      410        420        430        440        450        460        470
```

8. AGAP007630-PA BR4 2L AGAP000669-PA X 65.278 9.82E-34

```
                   10        20        30        40        50        60        70
AGAP00    MSDPVARPMKFPYTFSAKLAQFPIQHYFKNQWIWRYYFIAFGVSIPLFYKIHKLANSPANQAKWAESKRKE-HEEHH
             :.:  .:.:::..  :.:::: .::.::::::::.:::.::.:.:::::.:.::: :::.  :::::::.  .  .::
AGAP00 MSEEQSVSRTVKYPYTYTQKIAQFPYKHYYKNQWIWRFYFISFGLSLPLFYKLHSLANVPANKEKWAESKRKQLQPDHH
                 10        20        30        40        50        60        70
```

9. AGAP013199-PA BR5 2R AGAP000721-PA X 67.816 1.46E-178

```
                10        20        30        40        50        60        70        80        90       100
AGAP00 MPGYMFNIDGGYLEGLCRGFKCGILKQADYLNLVQCETLEDLKLHLQGTDYGQFLANEPSPLAVSVIDDKLREKLVIEFQHMRNHAVEPLSTFLDYITYS
       : :  .::::::::::.:::::: .::::.:::::.::::::::::::.::.:.:.:: ::::: ::..::.::..::: ::.::...::.::.::. ::::::::
AGAP01 MSGCLFNIDGGYLEALCRGFKSSILKQSDYLNLIQCETLEDLKLQLQSTEYGPFLANETSPITVSIIDQRLRETLVVEFSYLRNQAVQPLAEFLDYITYS
                10        20        30        40        50        60        70        80        90       100

               110       120       130       140       150       160       170       180       190       200
AGAP00 YMIDNIILLITGTLHQRPISELIPKCHPLGSFEQMEAIHVAATPAELYNAVLVDTPLAPFFVDCISEQDLDEMNIEIIRNTLYKAYLEAFYDFCKNIGGT
       ::::: :::::: ::.::.  ..:   :.::: ::::::::...  .:..::::.:.:.::.::: .:   :::.::.::::.:::::::.::: ::..::.::.
AGAP01 YMIDNTILLITGMLHKRPVLDVIAMCNPLGLFEQMEAINMTLAPSDLYNAILIDSPLGPFFDNCCWEQDMDELNIEILRNTLYKTYLEKFYNLCKEIGGA
               110       120       130       140       150       160       170       180       190       200

               210       220       230       240       250       260       270       280       290       300
AGAP00 TADVMCEILAFEADRRAIIITINSFGTELSKDDRAKLYPRCGRMNPDGLAALARADDYEQVKAVAEYYAEYAALFDGSGNNPGDKTLEDKFYEHEVKLNM
       ::.:::::::::::::::::::::..:: : :..  ..::: :::. :.:: ::.::.:::::. ::  ::::..::.   .:  :  .::::: .:.::.
AGAP01 TANVMCEILAFEADRRAIIITINALGTALPKEEYVHLYPCCGRLYPEGLLALGRASDYEQVRLVASRYAEYGTLFEDLNNLDG-WSLEDKFSGYEAKLHA
               210       220       230       240       250       260       270       280       290

               310       320       330       340
AGAP00 YGFMQQFHFGVFYSYLKLKEQECRNIVWIAECVAQKHRAKIDNYIPIF
         .::::::::::::::::::::: :::::::::..:..: :: ::: ..
AGAP01 RSFMQQFHFGVFYSYLKLKEQEFRNIVWIAECIVQNQRNKIRNYISLL
          300       310       320       330       340
```

10. AGAP028116-PA BR5 3R AGAP000951-PA X 44.053 1.50E-52

```
          10        20        30        40        50        60        70        80        90
 MSCNDKNDGISKEEWQSRLETFPFKQDDINKLIMNYLVTEGFKEAAEKFQAESGVTPSVDLNSLDNRIQIREAVQNGFIQEATHLVNQLHPELLDNDRY
           : :::..::. .:  ....:.:.:.:...  .:::::.::  ::.:..:.::  .:: : .. :....   ::  :....  :  : ..:...
MNPTKPKPETPSVEEWNKRLQGYPPMKESLNQLIVNYFIGAGFKEAADKFIQESNTTPTVNECSIDFRSRIIETIRQGNALEAMDLVDSFAPGLAESDRW
         10        20        30        40        50        60        70        80        90       100

100       110       120       130       140       150              160       170       180       190
 LYFHLQQLHLIELIRAGKIEEALTFAQTQISEAGESNPEVLNELERTLALLAF-----ETPQHSPFADLLGHSHRQKVASELNAAILKMEQQEQSSPRMI
 . ::::::::::::::::::::.  :...    ::     .  ..  .........        .    :  ..  ::  :.:.....::  ..  :::    ...:   :  ::
 MVFRLQQLQLIELIRAGSVEDALHYAQTVRWECPGRTQQVASEIERTMTLLLTLPQTRGTDPSSSFSTLLEQQHRDQVARTVNEALLLWDNRELPSARME
         110       120       130       140       150       160       170       180       190       200

   200       210       220
 NILKLILWAQTELDKKNVKYPKMMDLASATIEPK
 ................. ... .. .........
 HLFKLILWAQAELERKMVQFSKLKNMAEATFEVE
         210       220       230
```

11. AGAP009572-PA BR5 3R AGAP000541-PA X 95.385 7.12E-91

```
        10        20        30        40        50        60        70        80        90       100
MVRISVLADALKCINNAEKRGKRQVLIRPNSKVVIKFLTVMMKHGYIGEFEIVDDHRSGKVVVNLTGRLNKAGIISPRFDVALNDIERWTNNLLPSRQFG
::::::::::::::::::::::::::::::::::.::::::::::::::::::::::::::::::::::::::::::::::. ..:.:::::::::::::::
MVRISVLADALKCINNAEKRGKRQVLIRPNSKVVIRFLTVMMKHGYIGEFEIVDDHRSGKVVVNLTGRLNKAGIISPRFDTKVTDLERWTNNLLPSRQFG
        10        20        30        40        50        60        70        80        90       100

       110       120       130
YVVLTTSGGIMDHEEARRKHLGGKILGYFF
::::::::::::::::::::::::::::::
YVVLTTSGGIMDHEEARRKHLGGKILGYFF
       110       120       130
```

12. AGAP005098-PA BR7 2L AGAP000260-PA X 77.586 4.22E-29

```
         10        20        30        40        50
MAAWRAAGLNYINYSNIAARLLRKALKPELRAQAVRRDDSHIKFTKWQNGKPEKAITE
:::::::::::::::::::::..:::::::: :::.:.::..:::::.::::.::.:: ::.  ::
MAAWRAAGLNYINYSNIAARMVRKALKPALRADAARREESHIKMTKWKDGKSEKSATEA
         10        20        30        40        50
```

13. AGAP001701-PA BR8 2R AGAP000852-PA X 91.209 3.02E-58

```
               10        20        30        40        50        60        70        80
MADEKK---GSESEHINLKVLGQDNAVVQFKIKRHTPLRKLMNAYCDRAGLSMQVVRFRFDGQPINENDTPTTLDMEEGDTIEVYQQQTGGF
:::.::   .::::::::::::::::::::::::.:::::::::::::::::.:::::::::::::::::::::::.::::::::::::::::
MADDKKDPKSSESEHINLKVLGQDNAVVQFKIKKHTPLRKLMNAYCDRAGLSLQVVRFRFDGQPINENDTPTTLEMEEGDTIEVYQQQTGGGNRSNQ
          10        20        30        40        50        60        70        80        90
```

14. AGAP005558-PA BR7 2L AGAP000935-PA X 67.789 0

```
         10        20        30        40        50        60        70        80        90       100
MASLLKFSAGLRAYSRSHQLLFRRTKVSNAAEFRAALVNVPPTEVTTLDSGLRVASEDSGSQTATVGLWIDAGSRYENDSNNGVAHFLEHMAFKGTAKRS
:::::::.:...:    :.  :  :.  : :: ::.:: : : :::::::::::::::.  ::.:::::.::::::::.   .::.:.:.::.::::.:::
MASLLKLSTAVR---RQMLLAGSRAGSSYAAAFRSALSNEPATEVTTLDSGLRVASESVPSQVATVGLFIDAGSRYEDKHSNGTANFFEHLAFKGTTKRS
         10           20        30        40        50        60        70        80        90

        110       120       130       140       150       160       170       180       190
QTDLELEVENMGAHLNAYTSREQTVFYAKCLSKDVPKAVEILSDIIQHSKLGEAEIERERGVILREMQEVES-NLQEVVFDHLHATAYQGTPLGNTILGP
:. :: :::.:::.:.: :.:.:: : :.::::::::: ::::.:..:. .: .:...: : ::: :...:.. ::.::::::::.::.::: :.::. ::
QSALEQEVESMGAQLDASTGRDQTSFTARCLSKDVPKLVEILADVVQNPRLDDADVKRAREVILGEIEQVDAGNLREVVFDHLHSTAFQGTSLSNTVWGP
100       110       120       130       140       150       160       170       180       190

00        210       220       230       240       250       260       270       280       290
TKNIQSIGKSDLQQYIDAHYKAPRIVLAAAGGVRHGDLVRLAEQALGKVSSSVDGKAAALAPCRFTGSEVRVRDDSLPLAHVAIAVEGCGWTDQDNVPLM
..::.::   .:..  :...:::::::.::::::: ::...: .:::. :::. :. ::::   :.: ::::::.::::::::::::.::.:::::: .:.: . :
SSNIRSIKADDVRGYVNSHYKAPRMVLAAAGDVRQAELEKLAEKHLGKIESTFDGKAPQLSPVRFTGSEMRVRDDSLPLAYVAVAVEGCGVSDSDAMALS
200       210       220       230       240       250       260       270       280       290

00        310       320       330       340       350       360       370       380       390
VANTLIGAWDRSQGGGANNASKLAMASATDGLCHSFQSFNTCYKDTGLWGIYFVCDPLKCEDMLFNVQNEWMRLCTMVTEGEVERAKNLLKTNMLLQLDG
::..:::.:::. :::.:::::::.::: : :::.:.:::  :.::::::::: :::: ::::::::::::::::::::::.::::::::  ::: .: ::.:
VASALIGTWDRTFGGGVNNASKLAVASAHDKLCHNFESFNLTYRDTGLWGIYFECDPLMCEDMLFNVQNEWMRLCTMVTDGEVERAKRQLKTRLLAQLEG
300       310       320       330       340       350       360       370       380       390

00        410       420       430       440       450       460       470
TTPICEDIGRQMLCYNRRIPLHELEQRIDSVTAQNVRDVAMKYIFDRCPAVAAVGPVENLPDYVRIRSSMYWTRL
   :::::::::.:   .:: :::..:.::.:::::::::::.::::::::::::::::::::::::.::::::::::::
PHAICEDIGRQVLALGRREPLHDVERRIENVTAQNVRDVAMRYIFDRCPAVAAVGPVENLPDYMRIRSSMYWTRL
400       410       420       430       440       450       460       470
```

15. AGAP012315-PA BR7 3L AGAP001198-PA 2R 32.911 2.00E-27

```
                        10        20        30        40        50        60        70        80
             MKRLAFIIIPALIALGHSIRPPIIEGTEANLHEFPYQVSLQWNFNNGSRARHFCSGSIINQRWILTAAHCLEEYTKDGWFEVVAGVNN
                                .... . .. ..  ......... ..   . ... .........  .......   .  .   .......
GEIVLVKAICTRALPASFSASTNPIFSFADIKPDVANGGDAAENSFPYMVQIQ-QFMVVSYVHH-CGGTLVTSRCILTAAHCAVESLK---LRAIAGTVW
     20        30        40        50        60         70         80        90          100

90        100       110          120       130       140       150       160       170       180
IAHEEAGAQRRNVTRYEQHESY--DLSAIRYDIGVLQLSHPLDLTRNIKTMRLATKDTLIHQKIAKFAGWGSISKTWEDIYPDKLMKVNLILRTEEDCQT
   :  : ::: ..:   ::::  : ..  ::::.. ...:. .  .  ..     :      .      :.:.:.   .:  ::.:  :.   :.  ::::
RDSETLG-QRRPIVRLLAHESYVQDGTTQPYDIALALVEEPFVVDGRAIAVIALMPDYYDPPGVMDVLGFGKIDH--DDTLPDRLRVVECRLHDVEDCQK
110        120       130       140       150       160       170       180         190       200

 190       200       210       220       230       240       250       260
IGKIDETQICAGGYKNVSGCTADSGGPLTVTIDGEQMQIGVLSYGEKPCQARLPIVYSSVMYFHDWIQDAIKED
  ..  .....  ..... ........  .....  ....... .  .  .  ... ...  .....
--HPSEGTLCVGN-PGATACQGDSGGPVVGRIDGSDWLVGVVSFGMKSCGTG-PIICTDVHLYREWIGQRVFD
    210        220       230       240       250        260       270
```

16. AGAP011630-PA BR7 3L AGAP003713-PA 2R 58.252 2.76E-38

```
                  10        20        30        40        50        60        70        80        90
AGAP00       MKGLAVVAAVLLFACLVASQSNNYFWGVRDPRDVLLNRTIAVRSGTILQVKSIDLVYPLKGQVGRNISAISVVDQYTNGKGGYASLYAGGIGYNYTT
                 ..:: :..: ::.:..:..:::.: : : : :.:.: ...:...:.::: : :::::: ::::.:: : :.  .:.:::::. .:::: :::
AGAP01     MKFATVFVVVLAAVLGACMVTAQTHNYFFGSRIPYDSLVNQTTVIQSSSFLRVKSAYLDYPLKGQRGRNITAIYVYDRLGSGRGGYASITSGGIGKNYTR
                   10        20        30        40        50        60        70        80        90       100

            100       110
AGAP00     VHLKSQRGHGYNFIVEIYGR
           ..::.:::.:.:: ::::::
AGAP01     INLKTQRGNGMNFQVEIYGR
                  110       120
```

17. AGAP009948-PA BR8 3R AGAP006895-PA 2L 97.581 5.37E-87

```
                10        20        30        40        50        60        70        80        90       100
AGAP00 MATMHAHPGHPGGSRMQGPPPLTPWSPDKKHSDRERWVCIYPAYINRKKSRQEGRRIPKQCCVDDPSPQEIRDVLQALNMNVLVELKQYPRERSRELQCR
                      ::: ::::::::::::::::::::::::::::::::::::::::: :::::::::::::::::::::::::::::::::::::::
AGAP00                MQGSPPLTPWSPDKKHSDRERWVCIYPAYINRKKSRQEGRRIPKQYCVDDPSPQEIRDVLQALNMNVLVELKQYPRERSRELQCR
                              10        20        30        40        50        60        70        80

               110       120       130       140       150       160
AGAP00 GRIRVQLRNDDGAPLNSEYATRDSILLHLGKTIPLLKSRQAKPQEQSQAAASSSAGQKKGKGKRR
       ::::::::::::::::::::::::.::::::::::::::::::::::::::::::::::::::::
AGAP00 GRIRVQLRNDDGAPLNSEYATRDSVLLHLGKTIPLLKSRQAKPQEQSQAAASSSAGQKKGKGKRR
         90       100       110       120       130       140       150
```

18. AGAP011515-PA BR8 3L AGAP001676-PA 2R 98.67 0

```
                10        20        30        40        50        60        70        80        90       100
AGAP00 MCDDDAGALVVDNGSGMCKAGFAGDDAPRAVFPSIVGRPRHQGVMVGMGNKDAYVGDEAQSKRGILTLKYPIEHGIITNWDDMEKIWHHTFYNELRVAPE
       ::::::::::.::::::::::::::::::::::::::::::::::::::.::::::::::::::::::::::::::::::::::::::::::::::::::
AGAP01 MCDDDAGALVIDNGSGMCKAGFAGDDAPRAVFPSIVGRPRHQGVMVGMGQKDAYVGDEAQSKRGILTLKYPIEHGIITNWDDMEKIWHHTFYNELRVAPE
                10        20        30        40        50        60        70        80        90       100

               110       120       130       140       150       160       170       180       190       200
AGAP00 EHPVLLTEAPLNPKSNREKMTQIMFETFAAPAVYVAIQAVLSLYASGRTTGVVLDSGDGVSHTVPIYEGYALPHAILRMDLAGRDLTDYLMKILTERGYS
       :::::::::::::::::::::::::::::.:::::::::::::::::::::.::::::::::::::::::::::::::::::::::::::::::::::::
AGAP01 EHPVLLTEAPLNPKSNREKMTQIMFETFASPAVYVAIQAVLSLYASGRTTGIVLDSGDGVSHTVPIYEGYALPHAILRMDLAGRDLTDYLMKILTERGYS
               110       120       130       140       150       160       170       180       190       200

               210       220       230       240       250       260       270       280       290       300
AGAP00 FTTTAEREIVRDIKEKLCYVALDFEQEMQAAAASSSSEKSYELPDGQVITIGNERFRAPEALFQPSFLGMESTGIHETVYNSIMRCDVDIRKDLYANSVL
       ::::::::::::::::::::::::::::::::::::::::::::::::::::::::::::::::::::::::::::::::::::::::::::::::::::
AGAP01 FTTTAEREIVRDIKEKLCYVALDFEQEMQAAAASSSSEKSYELPDGQVITIGNERFRAPEALFQPSFLGMESTGIHETVYNSIMRCDVDIRKDLYANSVL
               210       220       230       240       250       260       270       280       290       300

               310       320       330       340       350       360       370
AGAP00 SGGTTMYPGIADRMQKEITSLAPSTIKIKIIAPPERKYSVWIGGSILASLSTFQTMWISKHEYDEGGPGIVHRKCF
       :::::::::::::::::::::::::::::::::::::::::::::::::::::.:::::::::::::::::::::
AGAP01 SGGTTMYPGIADRMQKEITSLAPSTIKIKIIAPPERKYSVWIGGSILASLSTFQSMWISKHEYDEGGPGIVHRKCF
               310       320       330       340       350       360       370
```

19. AGAP003091-PA BR8 2R AGAP008667-PB 3R 33.333 3.98E-14

```
        590       600       610       620       630       640       650       660       670       680
VVETSTEVLSGAYVDFKQIVIKPNMTAAVDLQSDAVFAGIKDRVAENEAKAKAINAVFLYKITSGGKVAKEWVLDLKNAKVYEGPVQGGGKADTTMTIAD
                              :.:: ::  :  :. .  .  .  . .. :. ..: ..: :.: ::::: .:. ::      : :..:.:. :
                              MALKSDPVFERIAKRLESIDPNNRQVQQVYKFRIQQNGTVVKTWVLDLKAVKLTEGD----GPAEATLTMED
                                      10        20        30        40        50             60

        690       700       710       720
GDMIELALGKLQPQTAFMKGKLKIAGNIMLAQKLAPLLKTEAKL
  :. :. :  .   . :. . :: . :.. :    : :.. .  :
DIMFALGTGAMPAKEALAQDKLDVEGQVELIFLLEPFIASLKK
70        80        90       100       110
```

20. AGAP007107-PA BR8 2L AGAP010239-PA 3R 59.942 3.99E-145

```
         10        20        30        40        50        60
MGKDYYKTLGIPRGSTDEDIKKAYRKLALKYHPDKNKSPGAEEKFKEVAEAYEVLSDKKK
::::.:: ::.  ....:..:::::::::::::::::::::.: :::.::::::::::::::
MGKDFYKILGVSKNASDDEIKKAYRKLALKYHPDKNKAPQAEERFKEVAEAYEVLSDKKK
         10        20        30        40        50        60

         70          80        90       100       110
REMYDKFGEDGLKGRASN--GTSNSSQNFTYEFHGDPRATFAQFFGSNNPFGSFFDMHND
:..::..::.:::: :..    :....: .: :.::::::::::::::...::. ::    .
RDIYDQYGEEGLKGGAGGMPGAGGQSGQFQYNFHGDPRATFAQFFGTSDPFSVFFGTDGG
         70        80        90       100       110       120

120       130       140       150        160       170
-SLFNSSIFNDDDFFTPFSGLGNRHGL--GGAFRSHSFNVH-SPLKKEKVQDPPIEHDLY
 ..:..  .    :  :  :   :.:  :.   :     ::::::.::::: :: .:.:.::::::::::
GNIFHQEM--DGDPFG-FDGRGGSVGGFPGGAFRSQSFNVHGSPQRKQKLQDPPIEHDLY
         130       140       150       160       170

   180       190       200       210       220       230
VTLEEIYHGCVKKMKISRRVLQPDGTSKKEDKCVSISIKPGWKSGTKVTFQKEGDQTKGK
:.::..   :: ::::::. :.   ::...::.: .::..::::::.:::.:: .:::   ::
VSLEDVNAGCQKKMKISKMVMGQDGSARKEEKILSINVKPGWKAGTKITFPREGDQIPGK
180       190       200       210       220       230

   240       250       260       270       280       290
IPADIVFIIRDKPHVWFRREGSDLRYTARLTLKQALCGVIFEVPTMTGEKLRISTKQEII
.:::::::::::::. :.::::::..:::...:.::::::.. .:::..:: : ::: :..
VPADIVFIIRDKPHAHFKREGSDIKYTAKISLRQALCGTVVKVPTLSGETLTISTAGEVV
240       250       260       270       280       290

   300       310       320       330       340
KPNTVKRIQGYGLPFPKEPSRKGDLLVAFDIKFPDKLSTSEKEMLNDMLPNS
::.::::.:. ::::::::::.:::.::::::.:::..: : ::.: :..:
KPHTVKRLQNRGLPFPKEPSRRGDLVVAFDIRFPDQVSPSTKEILADLFPMDVA
300       310       320       330       340       350
```

21. AGAP002346-PA BR9 2R AGAP000655-PA X 99.291 4.22E-93

```
        10        20        30        40        50        60        70        80        90       100
MAPSRKNKVVKEEVQVSLGPQVRDGEVVFGVAHIYASFNDTFVHVTDLSGKETISRVTGGMKVKADRDEASPYAAMLAAQDVAEKCKSLGITALHIKLRA
::::::::.:::::::::::::::::::::::::::::::::::::::::::::::::::::::::::::::::::::::::::::::::::::::::::
MAPSRKNKVAKEEVQVSLGPQVRDGEVVFGVAHIYASFNDTFVHVTDLSGKETISRVTGGMKVKADRDEASPYAAMLAAQDVAEKCKSLGITALHIKLRA
        10        20        30        40        50        60        70        80        90       100

       110       120       130       140       150
TGGNRTKTPGPGAQSALRALARSSMKIGRIEDVTPIPSDSTRRKGGRRGRRL
::::::::::::::::::::::::::::::::::::::::::::::::::::
TGGNRTKTPGPGAQSALRALARSSMKIGRIEDVTPIPSDSTRRKGGRRGRRL
       110       120       130       140       150
```

22. AGAP005197-PA BR9 2L AGAP000145-PA X 48.252 1.71E-36

```
          10        20        30        40        50        60        70        80        90
  MALKRINKELQDLGRDPPAQCSAGPVGDDLFHWQATIMGPPDSPYQGGVFFLTIHFPTDYPFKPPKVAFTTRIYHPNINSNGSICLDILRSQWSPALT
     .:: :: : : ..:     :: :  .. ..  .... ..:: :::..::.: : . .: :::.. ::: : :.::::::.  :  ::::::...:::::
MAALPRRIIKETQRLMQEPVPGISAIPDEQNARYFHVIVFGPEDSPFEGGLFKLELFLPEDYPMSAPKVRFITKIYHPNIDRLGRICLDILKDKWSPALQ
        10        20        30        40        50        60        70        80        90       100

100      110       120       130       140
ISKVLLSICSLLCDPNPDDPLVPEIARIYKTDREKYNELAREWTRKYAM
:  ::::: .::  :::::::. ..:...:... .  . :.:::..::
IRTVLLSIQALLSAPNPDDPLANDVAELWKVNEAEAIRNAKEWTQRYANVDN
       110       120       130       140       150
```

23. AGAP005981-PA BR9 2L AGAP000008-PA X 56.553 5.90E-147

```
               10        20        30        40        50        60        70        80        90       100
AGAP00 MVFETKFYDILGVAPSCTPDELKKAYRKLALKYHPDKNPNEGEKFKQISMAYEVLSDPEKKAIYDEGGEAAIKQGAGGGGGGFHSPMDIFHMFFNGGFSG
       :: :: :::.::: :.:.:..:::::::::.:::::::::::::.:: ::::::::::::::::::::::::::::: :.:::::::::::: :..:::..:
AGAP00 MVKETGFYDVLGVKPGCSPEDLKKAYRKLAMKYHPDKNPNEGERFKAISMAYEVLSDPEKKAIYDEGGEAAIKQG-GAGGGGFHSPMDIFDMLINGGMGG
               10        20        30        40        50        60        70         80        90

              110       120       130       140       150       160       170       180       190       200
AGAP00 RKNERQTSNVIHTLSVTLEELYTGTKRKLALQKNVICESCEGIGGKRGASQKCAPCRGTGVITKVQKIAPGLVQQYEERCRNCRGLGETIDDKDRCKECN
        . :..  ...: :.:::::::.:. :::.:::.:::. :.:::::::. .::.:: :::..:::..: ::..:: .  :: :.: ::..:.: .::.:.
AGAP00 GRREQRGRDLVHRLTVTLEELYSGATRKLSLQKSVICDGCDGIGGKRGTVHKCVPCNGTGILTKVHHIMPGFMQQNKVPCRACQGQGEVFDEKHKCKKCD
     100       110       120       130       140       150       160       170       180       190

              210       220       230       240       250       260       270       280       290       300
AGAP00 GRKTVRMRKLLEVEVYPGMVDEQRIVLKGEGDQEPDCRPGDIVLVVEEKSHPVFKRNGQDLLVFLHLQIAEALCGFRKVIKTLDARDIVIQSCPGEVVKH
       :.: :: .:.:.:..  :: : :.::..:::::::  .:::::...::. ::::::.:.::.. ..:...::::::.::: ::: :..:: : ::::.::
AGAP00 GQKKVRDKKILDVHIEKGMRDGQKIVFSGEGDQEPGLQPGDIVIALEERPHPVFKRSGKDLMMEMRLELSEALCGFQKVITTLDKRSLVITSMPGEVIKH
     200       210       220       230       240       250       260       270       280       290

              310       320       330       340       350       360       370            380       390
AGAP00 LSTKCVYGEGMPLMNDPTEKGRLIIQFVVGFPDSLPPEVVPEIRKYLPTPQP-DPIPEDHETVEMMDFSLEE-----YQKASEEDEGSDESGSYSRFNHG
        . ::.. ::::  ..: ::::.:: : :::::: :..  ...::: :.: . ::.: : ::..... :.     :..: ::::           . :
AGAP00 SAFKCIMDEGMPQWKNPFEKGRLIMQFRVVFPDSLPGEAAKLLEQYLP-PKPAEEIPQDVEMVELVELDPEQESRNQYKNAYEEDE-----------EDG
     300       310       320       330       340       350       360       370       380

          400
AGAP00 GGQRVHMQTCNSS
       :   :..: : .:
AGAP00 GTPGVRIQQCATS
       390      400
```

24. AGAP009444-PA BR8 3R AGAP004723-PA 2L 35.676 3.18E-32

```
              10        20        30        40        50        60                         70
      MSNKKLKPEEPVEEVNEFSVEKILDSRVVNGKVEYFLKWKGYSSEENTWEPEENLDCDDLIQAFKESR------------------KKKEAKEE
                      :. ::::.: :  .:::::.::::::.: .:.:::.::::: .::.::..::                  :::..  :
MGRTKEPKNTNGNSGNDSDTEEEYVVEKIVDRRERKGKVEYLLKWKGYDSGSNSWEPRENLDCPELIKAFEQSRTDAAKKDGAAAGKKRTTTKKKRTGGE
        10        20        30        40        50        60        70        80        90       100

                       80        90       100       110       120       130       140       150
SSG------------------RKSKLKDSSEEPKAVPAKRKATSDKKVGFDRGLIPEEIIGATDEHGKLMFLMKWKNAANADLVPAEQANVKCPQIVIKF
:..                  .::: .:.. .:: . ..    . .. ::..: .::.:.:::.  ..:.::..::.  .:.:: ...:.  :::.:: :
SENDNDGSDDEGRDDNASTKSNKSKSNDDGGDPKQADGE---AVEELNGFEKGYVPEKILGATEGDNELLFLIQWKDKDKAQLVKSKEARKHCPQLVIDF
       110       120       130          140       150       160       170       180       190

160      170
YESRLTWQSAEAKNDTRDD
::.:: ::...
YEERLIWQTVDQSQVD
200      210
```

25. AGAP007024-PA   BR9 2L   AGAP000498-PA   X   93.846   6.87E-92

```
                10        20        30        40        50        60        70        80        90       100
AGAP00 MALNPQYEEIGKGFVTQYYALFDDSTQRPTLVNLYNAELSFMTFEGQQIQGAAKILEKLQSLTFQNITRVLTAVDSQPMFDGGVLINVLGRLQCDDDPPH
       ::.:::::::::::::::::::::::::::.:::::::::::::::::::::::::::::::::: :::::::::::::::::::::::::::::.::::
AGAP00 MAINPQYEEIGKGFVTQYYALFDDSTQRPSLVNLYNAELSFMTFEGQQIQGAAKILEKLQSLTFQNIKRVLTAVDSQPMFDGGVLINVLGRLQCDEDPPH
                10        20        30        40        50        60        70        80        90       100

               110       120       130
AGAP00 AYSQTFVLKPIGASFFCAHDIFRLNIHNTA
       ::::::::::.:..::::::::::::::.:
AGAP00 AYSQTFVLKPLGGTFFCAHDIFRLNIHNSA
               110       120       130
```

26. AGAP010182-PA   BR9 3R   AGAP000200-PA   X   44.146   1.44E-163

```
                    10        20        30        40        50        60        70        80
              MSLHEWYRRRERGLPARIGDTDMIYRTIFRSLQPRALSAELSHPYRCGQDSGAICNLEFSPDGTLLAAACEYKSIVLFDPLAEKQVSAV
              : .:.:  ::::::  :.::.: :.:::.:::.:. .   . ..      .: :.  ::::::::::::.:::. :::::::::.: :. ::
MQTSGSTSNKFMPIHQWCGRRERGLGPRFGDADTIHRTIYRSLEPKKIWNDPGRFPGIVRDYGGAFNLEFSPDGTLLVAACQKKSIVLFDPLTEIQICAV
         10        20        30        40        50        60        70        80        90       100

90       100       110       120       130       140       150       160       170       180
SNAHDGSVNCIKFVDSRTFASCSDDTTVALWDARNLSTKLRTLHGHSGWVKNIEYAKGAGVLLSSGLDGLVYAWELNNSTEQGCTYQRLLYMPGLMRCRL
.:::    :::::.: . ::.:::::::::: :::.:::::::.:::::::::::.:    .:::::.:: ::.::.:: :: : .:.:.:.   ::.: ::
KNAHTECVNCIKFIDHQIFATCSDDTTVALWDIRNLTTKLRTLNGHSGWVKNIEYSKRDRMLLSSGFDGSVYGWEINNYTEYGSVYRRVLHTSGLLRSRL
        110       120       130       140       150       160       170       180       190       200

190       200       210       220       230       240       250       260       270       280
APDESRLVLCTGTGYLMLVHDLDLASLAGDLVDFRPNIQRLNLMRKQRVPLGARKTPAVSHRRKRNRIEFVSDFPPGDDAEMISGLTLHPQGWCALSRNI
.:::::::  ..:::...::::::.:::::  : ::. ::   . .: .:..::       .::.::::.:.::::... ::::.:..:::::.:.::.
SPDESRLVLSMSSGYLIIIHDLDLANLAGDLEGFYPNVYRLMQVGRQLIPMAARFRHLFYSKRKKNRIELVTDFPPNNNPEMISSLAIHPQGWCTLTRNV
        210       220       230       240       250       260       270       280       290       300

290       300       310       320       330       340       350                                 360
SYDEKTEWSVVHDIQSWSENEDESDTEKQEPDELNTEDSQEPEQEDVPQQERSNCTRSMEGFGRDPK-------------------------REPSETV
: :: :::::::::     :   : :..: .:  .: .:. ..::  : :.:.  .:  . .:::.                          ::   ..:
SSDEMTEWSVVHDIQ-----ELPHDEESDEDEEELAEREQNRREEDNDQAEESS--NSGVNVARDPQPGTSGMSGRSARGSELGGMFRLNVTMREMMSSV
        310             320       330       340        350       360       370       380       390

    370       380       390       400         410       420                                  430
LT--EATWCNGATERNVINNAFPSHSGAPCPRLAEVPLQPTIRLSPP--AKGGRAGSAWSEPVASK------------------RTAGHSTDGTGT----
.   : :  .. .. :. .:. :. ::.      .         : : :::  :.   .:: . ..: :..                  :. . :::   .:
ESHREPTVGSNEANANAEGNVDPQPSGSRSNLV-------TSRSSPPHEAQHVEAGPSGQQPPAANVRRQNGRGRRVMITRRNLRSENASTDVWATEVTF
   400       410       420                430       440       450       460       470       480

                                                 440       450       460       470       480
-------------------------------------------------RLLYYIQESNKVEGYMREACFSPDGRVICSPHDDYGVRLLAFNEQCNEMR
                                                 :.:::.::.   .:..::.::::::.::::::  .:  ::::::.::::..
RERKNMPSPQVRNNRGQIYGIVSGVSSSASTGYTGAGRKRANEKICKNVPRMLYYMQETITDQGFIRESCFSPDGRIICSPHD-HGFRLLAFNENCNELQ
 490       500       510       520       530       540       550       560        570       580

490       500       510       520       530       540
YARQCLASGSEPAPLRELRYKPCYPHVVISSQFSPRFPLLVTGCLLGNVVWNLPVLS
.:    .   ...    :.:.. : :.   .:.:::::::::::::::::::..::.  :.:
HATG-IWRNEKAQQLHEVKRKQCHSSLVVSSQFSPRFPLLVTGCLLGKIVWHQPALY
   590       600       610       620       630       640
```

27. AGAP011362-PA   BR9 3L   AGAP028592-PA   X   36.334   1.67E-58

```
        10        20        30        40                     50        60        70        80
MPPKNKKCSICGTNSTVRWIILSRKTLCDGCYDVQCNPPLEPRQEDGPD-------------EPVAGPSGLGRKSETILATGPVQSNPADDGIFLKPTFI
: ::  .::::::: .:::: .::::::::: .:::: :::::::: .::              .  ...  ...  ....  :      ::   .  .::      ::::
MSPKVQRCSICGTLETARWYLLDRRSICSDCHDVQLNPPLDPREERSPEPENRPQQYEDLMEQDASNASAVQHSSERALLECPVTPKKVD---VLQPSTE
        10        20        30        40        50        60        70        80        90

 90       100       110           120       130       140       150         160       170       180
PPRPAKIARVNSATLDGATTTSSGS---SSSSTTTTNTTATSATHSSSRSTTPSKPASRNKARKSFPSKP--KLTDETKRPKSVPKVFHNDYWYEVGDIV
      ...   .  .  . .:        ..       .   :      .   .   .:.   .:   :  .:  .  .:.:... .:        :    ::      ..: ::.... ::.:::::
GQADLELGADGFVGMDEQEELHDSVPPVGMFSPRRLRRRVCPVTRVPARRGT-KKNGRTTKSRRALTKKQPTKAPRETASTRTVSKVLQDNVWYQVGDIV
100       110       120       130       140        150       160       170       180       190

     190       200       210       220       230       240       250       260       270
SLVDTKDKTYYAQIRGLVVDVFNEKNAILTWLVPTTASPNPNEGFDPATYHIGPDEDKMRNLSEMQFVMHAPSGYYLNRSDPYPRQNVTGP---VERDGR
:..::::.:::::::::.::..:::.:.::::.:.:.:: ::::::::::  ::::.   :.:.  :.:::.:: .:.:.:..:::: .   ::      .. .:.:
SMLDTKDNTYYAQIRGLIVDAYNEKSAVLTWLLPSTVSPPPNEGFDPATYMAGPDEEAPRRLTYMHFVMNAPVNYFLDRNEPYPRPECYGPSNTTQCDNR
 200       210       220       230       240       250       260       270       280       290

280       290       300
STEPPLYDWANICQLHLGERPVSK
.      : ::..
N-----YVWATMGR
      300
```

28. AGAP009660-PA   BR9 3R   AGAP007722-PA   2L   41.27   2.41E-41

```
         10        20        30        40        50        60        70        80        90       100
MAPNAAELKVKPASDSTGINAILLGPPGSGKGTQAPLLKEKYCVCHLSTGDMLRAEIASGSKLGAQLKKVMDEGKLVSDELVVDMIDSNLDKPECRNGFL
                        ::..: ::::::: .  . . . . :.:.::.:::.: . ..::      : . ::::: :  ..  : :.:.. .  ....:
            MASGKLFRAIIMGAPGSGKGTVSGRIVKAFSLKHISSGDLLRANIEKRTELGLIADKYIREGKLVPDIYITKCILSELEQIRS-HSWL
                    10        20        30        40        50        60        70        80

        110       120       130       140       150       160       170       180       190       200
LDGFPRTVVQAEKLDNLLEKRNTGLDAVIEFGIDDSLLVRRITGRLIHQASGRSYHEEFAPPKVPMRDDETGEPLMRRSDDNAQALVKRLESYHRQTKPL
:::::::  ::. : :   ..:   .:.::.. .   .....:: .: .:   ::: :.   :   ::.: ::: ::::: .: ::.   :. :::: :    :.:.
LDGFPRTREQADDLWN--QER---IDSVINLDVPFEVIIERIQSRWVHLPSGRVYNVGFNDPKTPGRDDVTGEPLSQRPDDTPVAVRKRLEVYDACTRPI
 90      100             110       120       130       140       150       160       170       180

        210       220       230       240
ADYYALRGLHFRVDAAKSASDVFANIDSIFTKQRAHRLGF
 .:.   .:.
NEYFDKKGVLVTFKGSTTDEIWPHVRKYLEAKIQ
     190       200       210
```

29. AGAP009701-PA   BR9 3R   AGAP006997-PA   2L   33.266   6.37E-156

```
10        20        30        40        50        60        70        80        90           100
SRVLSRHQLVRQPTNQVRYVSVGRAGPSSLEIRRQFIDYFTVKHNHRLIRSSSLIPFNDSTIAFVNAGMNQFKSVFLGTAERPCQ-----RAVNSQKCVR
                     .::  :.:.:   :. :   ..:::.::..: :. :.::::::: .::::..      .        :..:.::::.:
                     MDTSLTASQIRSIFLDFFKEKE-HLYVHSSSVIPLDDPTLLFANAGMNQFKPIFLGTVDPNSDMAKWVRTANTQKCIR
                             10        20         30        40        50        60        70

     110       120       130       140       150       160       170       180       190       200
VGGKHNDLSVVGTDSYHHTFFEMLGNWSFGDYFKREACEMAWDLLRNVYRIDTDRVYVTYFGGDAKLNLPADEECRQIWRSLGIPPERVLPFGARDNFWE
.:::::::. :: : :::::::::::::::::::.: :   ::.:: .   ..   .:.:::::::    . .:   : :::.:: .::.   :..:: . .:::::
AGGKHNDLDDVGKDVYHHTFFEMLGNWSFGDYFKKEICTWAWELLTERLKLPKERLYVTYFGGHPESGLEPDLECREIWLKLGVKEEHILPGSMKDNFWE
 80        90       100       110       120       130       140       150       160       170

     210       220       230       240       250       260       270       280       290       300
MGNSGPCGPCTEIHLDLSGEYRNTKARQHLVNAGVPDLTEIWNIVFIQYNRSLDDGTIRNLPQRHVDTGMGLERLVAHLQHKQSNYDTDLFEPIFRRIQK
::..:::::.:.:.:   :        ..  .:::    ::. ::::.::::::. .:.... ::..:.: :::.:::. .: :.::::::.: :.:   :::
MGETGPCGPCSELHFDRIGG----RSVPELVNMDDPDVLEIWNLVFIQYNRE-QDSSLKLLPKKHIDCGMGFERLVSVIQDKRSNYDTDVFMPLFDAIQK
180       190           200       210       220        230       240       250       260       270

     310       320       330       340          350       360       370       380       390
ATKKEPYRGSFLSTDSHYELDTAYRIMADHSRMITACLADGMFPS---QNHKLRRIIRKSLALATRTFN-HPQLLRETVPCVVEILSTVYPEMGRNLPTV
.:    :.:    . ::    .: :::..:::.: :: .:::: ::.     ... ::::.:...  ::. .:   :  ..    :   ::..:. ..::. ..   .
GTGAAAYQGRVGADDSD-GVDMAYRVLADHARTITIALADGGFPDNTGRGYVLRRILRRAVRYATEKLNAKPGFFATLVDTVVQLLGETFPEVRKDPQHI
      280        290       300       310       320       330       340       350       360       370

400        410       420       430       440       450       460       470       480       490
LQII-EHEQHLYSALRTKRSAETSALLQQFPQLEESEALEHPGLADAIKELAQTKPTHLNGAHIHKLYDTYGLDEELLVKLGEMMHFSLDFRDYERYVR-
 ..: :.::.. ..:   :.                     :  .: .:...:   . :    .::::::.  .: . ..:   ....:.. ::.  .
KNVINEEEQQFLKTLTRGRNL---------------------LNRTIAKLGNSKV--IPGDVAWRLYDTYGFPIDLTLLMAEEKQMTIDMEGYEKAKHE
      380       390                       400        410       420       430       440

   500       510       520       530       540           550       560              570       580
--VLKDGHKHELATKLQTRLSAYENLRESLDSATLKPTRAERRYNYAFNADK----GTYEVAPCRARVSLLL-------EDEARDQWHIVTDQSNFYCES
  ....: . .  . .. ..  . . ..:.:    ::       .  ..: ..:..   . :   :: ...   :           : .: ..  .. :..:::.::
SYIISQGKEKSKTATIDLDVHGISELQERKVPAT------DDSFKYRYKAESIDPLAQYVFEPCTGKIVALRFNNAFVEEVQAGQECGVILDRTNFYAES
450       460       470       480               490       500       510       520       530       540

    590       600       610       620       630       640       650       660       670       680
GGQQSDTGRLTVEAGGSAGGQTFEVTGVTDHNGFVMHSIKRQPNVALSVGDTVVLQVDANRRTQLTLHHTATHLLNAVVRKVVALPMCQRSSSVSERHFR
:::  : : :.       :.   :.:. : ...:.:.:      . .   : ::: :   ..:. ::       .:.::: ::   . ::..       ::.: :   . .:
GGQIYDQGFLVKVNDESS---EFNVSLVYNRGGYVLHIGVVEGT--LRVGDEVHCHMDVVRRQLTMKNHSATHALNHSLLKVLGQDTDQRGSLVVPEKLR
      550           560       570       580          590       600       610       620       630
```

30. AGAP011054-PA   BR9 3L   AGAP007543-PA   2L   66.845   1.03E-93

```
 40        50        60        70        80        90       100       110       120       130
GSCHSFGGGHVYPQEAPRFVDHKLQYTKAVISRPAPAFEATAVVDGAFKKIKLSDYRGKYLVFFFYPLDFTFVCPTEILAFSDRVNEFKKLNAEVIAASI
                              ...:::::..::::.: ::.:.:::: :::.:.::::::::::::::::.:::::..:: . ...:::.:
                         MPVPELQKPAPAFSGTAVVNGEFKEIRLSDYLGKYVVLFFYPLDFTFVCPTEIVAFSDRADEFHEKKCQVIACST
                                  10        20        30        40        50        60        70

140       150       160       170       180       190       200       210       220       230
DSHFTHLAWINTPRKEGGLGKINIPLVSDITHSISKDYGVFLDDLGHTLRGLFIIDDRGVLRQITMNDLPVGRSVDETLRLVQAFQYTDKHGEVCPAGWK
::::::::::::::.::::...:::..: . .:..::::. .. :  .::::::::.: :::.:.:::::::::::::::::::.::.:::. ::::::.::
DSHFTHLAWINTPRKQGGLGELKIPLLADKSMKIARDYGVLQEESGVPFRGLFIIDDKGNLRQVTVNDLPVGRSVDETLRLVEAFRYTDEFGEVCPANWK
   80        90       100       110       120       130       140       150       160       170

240       250
PGQDTIVPNPEEKIKYFEKNH
::. :.: .:...  ::
PGSKTMVADPHKSKDYFNAVN
  180       190
```

31. AGAP008909-PA   BR9 3R   AGAP012962-PA   2R   61.783   1.81E-71

```
                                     10        20        30        40        50        60        70
                            MSDFVRDMRNHKIVPDVVPVPPESLLHVTYPGGLRVNLGNILTPTEVKHVPEVAWPEAEPDAYYALVLTDPDAPSRTAP
                            : ....:..:..::::::: :   . .::::.:  :.  ::.:::::.:: ::.: : .:.  : :.: .::::::::  :
MSICRLQTRASSIFTTSIRLFSSAIAKSMEKHEVVPDVVPVAPAEVAKVTYPSGAVVSEGNVLTPTQVKDVPKVEW-NADSGALYTLCMTDPDAPSRKEP
        10        20        30        40        50        60        70        80        90

80        90       100       110       120       130       140       150       160       170
 KFREWHHWLVVNIPGMDLAKGDTLSDYIGAAPPRKTGLHRYVFLLYRQNERIYYKESRLSNRSTQGRGKFSTHKFSEKYELGLPVAGNFFQAQFDDYVPK
 .:::::::: :::: :.:.:.::: :.:..::. ::::::::::.:.::  ..  . : ::.: :...:: :.  .::.:::.:: ::::::.::..:::::
 TYREWHHWLVGNIPGADVAQGETLSAYVGSGPPQGTGLHRYVFLVYKQNGKLTFDEPRLTNTSADNRGGFAIRKFAEKYQLGNPVAGNFYQAEWDDYVPL
00        110       120       130       140       150       160       170       180       190

80        190
 LYRQLGHFQNAF
 ::.:::
 LYKOLGA
```

32. AGAP009076-PA   BR9 3R   AGAP002370-PA   2R   33.697   1.85E-109

```
                               10        20        30        40         50        60
                            MLPKVPLPTVEQTMAEYVRVLQPIVTPQQLERTKSIIKQFSAT--IGPSLQEYLQAKREADDNWAYYYWLN
                              : . :.: . .:: . :: ..: :   . :  ::   ...:..   :: .::. :. .      :::   .::.
SPAANASGKMCPTSQQNYATAAAGGAGPLALQRQPVPKLADTMQKLVRSIEPHVDGDTLAGTKRAVEKFTVPGGIGQKLQSLLEQRAAQKDNWLADWWLR
   30        40        50        60        70        80        90       100       110       120

70        80        90       100       110       120       130       140       150       160
 DMYMDNPLPLPINSNPGMVMPPRKFTTVNDLAQFAAQLIDQLMDHKEMLEGGGLKQERATSREKGQPLCMAQYYRLLGSCRRPGDPRDS-QYLPPGGEQT
   ::.    :. . :.::.:.:   .. :...   :.::....   . .: ...:: .: :    .       :: :.:: ...:.:: ::  ::: :: :
 CAYMEYRDPVIVYSSPGLVFPRASYKTLDEQLQYAAKMVSAALAYKMLIDGGKIKPEMMGK----VPLDMSQYEKIFGTCRIPGKERDSVQYNP-----R
  130       140       150       160       170       180          190       200       210

170       180       190       200       210       220       230       240       250       260
 DAHVIVCCRNRMYCVPVKAGDRGRLNENELAAQLLHILNEAPGLPETEATVGILTTEPRPKWATDRELLLLEEQNARNIELIETALVVMCIDEPIPLSYN
 . :..:.:.:.: : .:: ..  : ..:... :.:  :   .   .   .  : .::::.. : .::      :  :. .   :   ..: :. :: :. ::. .: .
 SRHIVVACNNHYYRLPVFTAAGGIVSERQILAELKKIAAKEGN--SRAAPLGILTANHRDSWAQAYETLMADATNRASVESIQQALFVLSIDRELPQKQ-
 220       230       240       250       260         270       280       290       300       310

270       280       290        300       310       320       330       340       350       360
 ARGFNGSPAGAHYAGGRDESNMAHEM-IHGGGSACNTANRWFDKTMQLIICNDGTWGLCYEHSPSEGIAVVQLLEGILKRIDEAATAKDGPGTATADRLQ
               : :.   : .. ::::::.: .:::.:::.::... .:  :: :::::.::  .. .  . .:. :  .          :    : :. .
 --------------GTDHIVTASDLLIHGGGSAANGGNRWYDKTIQLVVAPNGINGLTYEHSPAEGQPIAVMTDFLLEYIGS------GKCLNTMDQAE
                 320       330       340       350       360       370       380              390

 370       380       390       400       410       420       430       440       450       460
 LQQSHLPPPERLEWIVRPEIERRLREAARSVDKRIEDLDFYVYRYKPYGKNFIKACQVSPDVYIQLALQLAYYKLYGHLVSTYESASTRRFLLGRVDCIR
 :. .  .    .:.. : : .. .. .:: .:: :...   ..   ::.:::.:. ..::: :::.:.: : :.:.:.     . .::::.:. .: :::. . ::
 LSAGAV----KLDFNVTPALRSEIDQAAGFVDKLAADIQMDYLHFTDYGKDFIKTQRMSPDSYIQMAIQYAFYRLHHVPGAHYESAQNRMYLHGRTETIR
         400       410       420       430       440       450       460       470       480

 470       480       490       500       510       520       530       540       550       560
 SASLEALEWAKAMCQGEGANVTLESDKEDDYSAEAAGDVKKKDHLRELFRCAAARQTEVMVQNILGYGIDIHLLGLREACREREGILHELFTDECYKIAN
 :.:.:.. .:.::        ::  ..   . ::            ...:    .    . . : :::.: :::::. ...:.     : ::..:.   . .
 SCSVESVAFARAM---------LEPKRDGRSKLEA------------MKAAINAHKAYVSMAIQGYGVDRHLLGLKLTAKENGLALPELYADQGLQKSA
490        500                 510                     520       530       540       550       560

 570       580       590       600       610       620       630
 CFLLSTSQVACSTNSFMGYGPVTPHGYGASYNPHPNEIIFCLSAFFTSDKTSASRFARSLQDSLDAMRDLLS
  . :::::::    ..:: :::.:   :::. :::   ... : .::: ..    :. .::   :::..:   :  ...
 HMRLSTSQVASRYDAFMCYGPLTQDGYGCCYNPKEDDMWFGVSAFRSNKDTDLARFRVSLQEALREMYEVLVVYGEKPKGKL
 570       580       590       600       610       620       630       640
```

33. AGAP001299-PA BR9 2R AGAP011723-PA 3L 30.238 1.77E-52

```
         10        20        30        40        50        60        70        80        90       100
MLKAVILIGGPEKGTRFRPLSLDTPKPLFPVAGKPIIQHHVESCVRIKELKEILILGFYPATQMQQFVSNMQNLYDVNIRYLQEFTALGTAGGMYHFRDQ
 ..:.::.::   :::.:::.:.:::::   :.:::. :..:. :.      . :: .. : : ::.   .: .  .    :.. . .:   ::::: .  .  .
 MRALILVGG--YGTRLRPLTLSTPKPLVEFANKPILLHQIEALVEAGVTQVILAVS-YRAEQMEAELSAQVERLGVKLIFSHETEPLGTAGPLA-LAKS
           10        20        30        40        50         60        70        80        90

        110       120       130       140       150       160       170       180       190       200
IRSGNPGAFFVLNGDVCADFPLQELYDFHRSKNEKALVSIMGTEATRQQAVHYGCLVLGKDEEVTHYVEKPRSYLSTLINCGVYVCSMDIFAKMGAVFHS
: . .   :::::.:. .:::..:: .:::   .... . .  .:     .   :: .. . .  .  ..:::....:. ::.:.:: . ...:..
ILAESTEPFFVLNSDIICDFPFKELEQFHRRHGREGTIVVTRVE----EPSKYGVVLYADNGCIKSFIEKPQEFVSNKINAGMYVLNPSVLARI------
  100       110       120       130           140       150       160       170       180

        210       220       230       240       250       260       270       280       290       300
KQQDYSMLNNGNGKDSGHIQWEQEILTPLAGTGKLYALPVNNWWSQIKTAGSAIYANRHYLALYKNAHPERLASAGLKTENDNANLVCNIVPDVHIHPTA
              .    . :.::.  ..   .:::. .:..: .:     . . .   ::.  .. .:.:: :.       :..: :.. :    :::
--------------ELKPTSIEKEIFPVMSHEQELYAFELNGFWMDIGQPRDFLTGMCLYLTSLRQRQPDRLYSGP-------AGFVGNVLVD----PTA
                 190       200       210       220       230       240              250       260

        310       320       330       340       350       360       370       380       390       400
SVHPTATLGPNVSIGPGVVIGPGVRVRESIILENAVIKDHSLVLHSIVGRGSQIGMWARVEGTPSDPDPNKPFAKMENPPLFNTDGRLNPSITILGYAVS
..   . .::::.:::.:::  :: ...  ::..:.::.:: .    :.:    .: :.:.:::                             :.::  :
KIGAGCRIGPNVTIGPNVVIEDGVCIKRCTILKDAIIKSHSWLDSCIIGWRCVVGRWVRLEGT----------------------------TVLGEDVI
        270       280       290       300       310       320                                330

        410       420
VPSEMIVLNSIVLPHKELSRSFKNEIIL
: .:. . .. ::::... :
VKDEIYINGGQVLPHKSIALSVPEPQIIM
      340       350       360
```

34. AGAP001403-PA BR9 2R AGAP012415-PA 3L 37.222 8.78E-37

```
                10        20        30        40        50        60        70        80        90
        MRGVWKLLFGRYLLVTNTISSGVLMLAGDVAAQEIERRQEKTTSASEGLERQRALNMTLVGLSQGPLHHYLYKWMDAYLPGATVRTVLKKIA
                     .:::.::.  :  :  .::.    ::.. :  ..   . .:.:.  :.. :.. : . :  :..:: .:: :.  ::::.
MIALAKWFRTATKTAFSKKYLLLTNVAISVSLSGVGDI----IEQHYEIYSGELAAWDRRRTRFMSISGMTVGVFCHGWYNFMDRRFPGRTIGLVLKKVL
        10        20        30            40        50        60        70        80        90

      100       110       120       130       140       150       160       170       180       190
IDQLVISPIFIVTYLYSAGLLEGASVRDCNAELRYKYWTIYTADWLVWPPTQFINFYLLSPKYRVLYINAITMLYNVFLCYIKHNDDFISEITQKMKSIK
::: : ::: :  .. . ..:. .: .. ..:.: :.  .:::.:.::::.:..:::.:  :::::: :.:.. :.:.  :. ....  .   :. .:
IDQTVASPIVIFLFFATLAVLKRSSWEEMRGEIRDKFIRLYTAEWVVWPPAQIVNFYFLPTKYRVLYDNTISLGYDVYTSYVINDESGATAATNAQKD
 100       110       120       130       140       150       160       170       180       190


QE
```

35.AGAP007020-PA BR9 2L AGAP011824-PA 3L 50 2.81E-75

```
           10        20        30        40        50        60        70        80        90
   MRIGATIPNFQADSTKGPIDFYEWIGDSWCVLFSHPADFTPVCTTELGRIAVHQEHFEKRNVKVLAHSVDDLKCHVDWVNDIKSYCPDIIGN-FPYPI
   ....:  .:::.:::.:.::::::.::::.::::::::.:::::::::. .:     .: ::::: .: : : .. :  :..::::.    .. ::.::
MSLNLGDPFPNFTADTTIGPIDFHQWIGDGWAILFSHPADYTPVCTTELAAVAKLVPEFTKRNVKPIALSCDTVESHRGWIEDIKAYGQLAAADPFPFPI
        10        20        30        40        50        60        70        80        90       100

100       110       120       130       140       150       160       170       180       190
IADPSRDLAVRFGMLDEKDKDNVELAQTVRALFIISPDKRVRLTMHYPTSTGRNVDEILRVIDSLQLTDRLKVIATPANWTPGTKVMILPSVSEEDADKL
: :  .:.:::...:::. .   .. :   : ::.:.:.  :..::.. ::.:.:::  ::::.:::.::::. .: ::::.: :: . :. :.:   ..   :
IDDSKRELAVKLNMLDRDEIGSAGLPLTCRAVFVIDAGKKLRLSILYPATTGRNFAEILRTIDSIQLTDKRRV-ATPADWMPGDSCMVQPTVPADQLATL
       110       120       130       140       150       160       170        180       190

200       210       220
FPNGIERVSMPSGNVYVRTTTDYE
:: :.. :..:::. :.: :
FPAGVDSVTLPSGKQYLRKTECPN
200       210       220
```

36.AGAP001384-PA BR9 2R AGAP008039-PA 3R 51.576 6.95E-130

```
             10        20        30        40        50        60        70        80        90
     MKQESSHKFSAGSDYHQTLSRLKTEFEKRYNDQKQASIASLTEYEILRTLGSGAFGTVKLIKRKASGEYFAMKILSKERIVYQKQLQHTTNEKRILQ
           .. : .:... .. :.. : .::.... .. .. :.: ..: ..:::.:.:: : ....:.. .:.:::::.:...: ::..:: :::::::
  MGNNATSANKKVDAAESVREFLDQAKEDFEEKWK-RNPTNTAALDDFERIKTLGTGSFGRVMIVQHKSTKDYYAMKILDKQKVVKLKQVEHTLNEKRILQ
          10        20        30         40        50        60        70        80        90

  100       110       120       130       140       150       160       170       180       190
  SIRFPFVVNLEICYKDNSYLYLVMPFVNGGEMFTLLRHSRRFSEAQAAFYGAQVALALEYLHGCNLIYRDLKPENLLVDYRGYVKMTDFGFCKLIKDRTW
  .: :::.:.:.. .:::: :::.:. .: :::::. ::   :::: .. ::.::..::.::::  .::::::::::::.: .::.:.:::::.: .: :::
  AISFPFLVSLKFHFKDNSNLYMVLEYVPGGEMFSHLRKVGRFSEPHSRFYAAQIVLAFEYLHYLDLIYRDLKPENLLIDSQGYLKVTDFGFAKRVKGRTW
100       110       120       130       140       150       160       170       180       190

  200       210       220       230       240       250       260       270       280       290
  TLCGTPEYLAPEIIQAKGYGKSVDWWSYGVLLYEMAAGYSPFYVNSSDQMVLFERICKGKYKFPKSFSTDLCSLVHHLLQTDLSRRYGNLRNGCDDIKQH
  ::::::::::::: .:::.:.::::. :::.:::::::: ::....  :  ..:.: .:: .::. :...: .:...:::.::..:::::. : .::: :
  TLCGTPEYLAPEIILSKGYNKAVDWWALGVLVYEMAAGYPPFFADQPIQ--IYEKIVSGKVRFPSHFGSELKDLLRNLLQVDLTKRYGNLKAGVNDIKGH
200       210       220       230       240         250       260       270       280       290

  300       310       320       330       340       350
  AWFKTINWYGLLNRELPAPYVPKLQGPGDASLFDVYDEQKLKIASKCLYAKEFADF
   :: . .: ....... ::..:. .::::.: :: :.:. :.:.:    ::::::.:
  RWFASTDWIAVFQKRIEAPFIPRCKGPGDTSNFDDYEEETLRISSTEKCAKEFAEF
  300       310       320       330       340       350
```

37.AGAP003106-PA BR9 2R AGAP009791-PA 3R 40.287 0

```
 170       180       190       200       210         220       230       240       250       260
  ISGCSADPFAPGCAGVTDESRRSSNICLTDECVRTASSLLAA--MDRTADPCKDFFQFACGTWNKMHVIPEDRSSISTFEVLADQQQAILKGVLEE----
                              : ::.:.  .    ::  :::: ::.:.:::.:.... ::.:.....:::.: .. .  .:: .: :
  TATEKAVERSEQLSRVRRSVGWMNDDRSSPESVRAAQVAIMKQYMDPDADPCDDFYQYACGNWDRVNPIPKDKAALDTFELLRESLDLVLKQLLLEGEPA
200       210       220       230       240       250       260       270       280       290

                                                 270
  -----------------------------------------PVNKEDNRATK-------------------------------------------KAKAF
                                           :.... .:. :                                            ::. .
  GLLDVENALSTVRSQPDGTKKSTSTTTTAASAGWPTTTVTGGPAQQQLHRVRKRGRAENRNRSGRAVQNKLIIRSAQVKRVRRKRELLINDDAEMKARHL
300       310       320       330       340       350       360       370       380       390

 280       290       300       310        320       330       340       350       360       370
  YKSCMNLEQIRLLDVQALRKSLKKLGGWPVIEKN-WTVPSTSIEHLLGKLTGEYDEPGLVELYVGADDKNSSMNIIQVDQLLLALPSRDYYLKESSEGDM
  . :::: : :.    .. ::   : .:::::::.: . :     : .   .: . :   .:..   :.   .:: : :::. ::.: ::   :.::.:::::.  ...   .
  FVSCMNYELIERRGLEPLRTLLHSLGGWPVLEPHTWDESSFDWLNLTAALR-RYNNDVLIVEWVGPDIKNSDENIVQFDQTSLGLPTRDYYLQPGNRKYL
400       410       420       430       440       450        460       470       480       490

  380       390       400       410       420       430       440       450       460       470
  KAYHRYMTQIAILMGADKDKAAEELQRIVEFEVRLANATLPEADRHDTSAIYTKITLPELQRRVPQINWKEYLQTTLGTVALHPNESIVSYAMPYLVELG
  .:.:...: ..  :. .  : :  .  ....:::..::: :    .:...:..: :. : .::..::::: .:: : .     .. .. .: .:: :. .:
  EAYRQFMLEVIGLLDVPADTARQATDEMIEFETQLANITSTPEERNNVSTLYRKLMLDQLQEEVPQINWTHYL-TIVTERPVNGSSFVVMFAMSYMRDLV
 500       510       520       530       540       550       560       570        580       590

  480       490       500       510       520       530       540       550       560       570
  KILRDTDRRIVHNYAIWRLVMSIMTHMIDDYQKERVEFRRKLLGIQSERNRWSQCVEWTNKKLGMAVGALFIRDNFNQESKETALTMIHTIREAFNELLA
  ... .:. ::: :: .::.:    .... : .    . .: . :.: . .   ::..::   .: ..:::::.:.:    :...::. .::: :  ...::.:.:.
  ELIDQTEPRIVANYLLWRFVRHRINNLDDRFLGAKQRFSNALFGRERNPPRWKNCVTQVNANMGMAVGAMFVRRYFDENSKRDTLTMTHELQDAFREILG
  600       610       620       630       640       650       660       670       680       690

  580       590       600       610       620       630       640       650       660       670
  DIDWMDDETRAVAKEKADAMNERIGYPDILTNADELEKEYVNLTIHGGLFLENILSILKWEAERNLQLLRKPVDKNKWATEPAVVNAFYNPNKNDIVFPA
     :.:  :: .:..:..::. ::::::.. . ..:. .:..: ::   ..:: :..:.     . . . : .::.:. : : ::::::.:. :::.:.:::
  RTGWIDMATRQLAEQKVNAMSLRIGYPDFILDPEQLSARYATLEIHPDRYFENTLNVLSHIRRTDQEKLGQPVNKTAWHTAPAVVNAYYSRNKNQIMFPA
  700       710       720       730       740       750       760       770       780       790

  680       690       700       710       720       730       740       750       760       770
  GILQPLFYSQNFPKSLNYGGIGVVIGHEITHGFDDKGRQFDKDGNMMQWWNNATIKTFRERAQCIIDQYSRYKIDEVGLYMDGRMTQGENIADNGGLKQS
  ::::: :: ...::..:::::::::::::.::::::::: ::.:::.  .::..  .:.  :.::: :...::.::.: :: . .::.  ::::::::::::.::.
  GILQPPFYHRHLPKAINYGGIGVVIGHELTHGFDDKGRLFDRDGNLYRWWSDRAIEEFHERAACLVEQYGRYRIAEVDVQLDGENTQGENIADNGGIKQA
  800       810       820       830       840       850       860       870       880       890

  780       790            800       810       820       830       840       850       860
  YRAYRKWVSQHGSE-----PDLPGLNMTHDQLFFLNYAQIWCGSMRPEDALTKIRSSVHSPGIIRVIGPLSNSRDFAEAYRCPLGSPMNPVSKCSVW
  . :: ::.. . ..        ::::::.:. ::::::.:::::::.:::: . .:....::::: .::::  :::.:::. :.::.:: ::: .:::::
  FLAYSKWLAAQTDRRVLEAETLPGLNVTRTQLFFLNFAQIWCGAMRPEATRNKLKTAVHSPGRFRVIGTLSNSEDFAREYHCPVGSTMNPPGKCSVW
  900       910       920       930       940       950       960       970       980       990
```

38.AGAP005423-PA BR9 2L AGAP008816-PA 3R 35.593 3.63E-46

```
        10        20        30        40        50        60        70        80        90       100
MFLTRSEYDRGVNTFSPEGRLFQVEYAIEAIKFGSTAIGISTPDGVVMAVEKRITSSLIEPSKMEKIVEVDRHIGCATSGLMADSRTLLDRARIECQNHW
     :.:::....:::.:.:.::::: ::.. ::::::.    : ::..:::. ....: :    ..::  .:.:.   : .:: ::.:.:..::....::.:
   MSSRYDRAITVFSPDGHLLQVEYAQEAVRKGSTAIGVRGKDVVVLGVEKKSVAKLQEERTVRKICLLDHHVVMAFAGLTADARVLINRAQVQCQSHK
           10        20        30        40        50        60        70        80        90

        110       120       130       140        150       160       170       180       190
FVYNERMSVESCAQAVSNVAIQFGDGDDTDSAMSRPFGVAILFAGIE-NGEPQLWHMDPSGTYIRFDAKAIGSGSEGAQQNLQEYYLPTMTIKE--AINL
.  .. ...:  .. ....  .        :.:   :::::.. :..:.. .: :.:.  .:::.: .. :.: : ... ... :.:.: :.  .   :  .:.:
LSEEDPVTLEYITRYIAELKQKH-----TQSNGRRPFGISCLIGGFDYDGVPHLYKTEPSGVYCEWKANATGRSAKTVREFLEEHYSPAAVSTEEGTITL
100       110       120            130       140       150       160       170       180       190

200       210       220       230       240
ALSTLKQVMEEKLNSTNVEVMTMTPKELFRMFSKEEVEEYINNMS
:. .: .:...  .. ..:: .:   : ..:.. . .:::....
AIRALLEVVQS--GQKSLEVAVMRRDEPMKMLDAQTIEEYVKKIELAKEEEAEKKKAKK
     200         210       220       230       240
```

39.AGAP006104-PA BR9 2L AGAP009128-PA 3R 37.5 1.09E-54

```
              10        20        30            40        50        60        70        80
       MALDFAAGCLGGCAGVLVGFPFDTVKVHLQTQN----HRNPLYRGTYDCFRKIVVREGVHGLYRGMSSPMAGVAVVNAIVFGVYG---NIQRRT
           : .: .:: . ::.: :.::.::.:::.      . ::: :: :: .: ..::: .:::.:::.:..::: . :. :   .:    ..:..:
MSENKSPIKYFLSGGFGGICTVLAGHPLDTIKVRLQTMPLPAAGQAPLYAGTLDCAKKTIAREGFRGLYKGMSAPITGVAPIFAVSFFGFGLGKRLQQKT
         10        20        30        40        50        60        70        80        90       100

 90       100       110       120       130       140       150       160       170       180
ANPDSLYSH-FLAGSAAGLAQSIVCSPMELIKTRLQLQDNLPRAAERFSGPMDCTRAIWRREGYRGIFRGLGITAARDMPGFSSYFVAYEYMVRCVANPS
 .  .  :.. : ::. .:.   . : .: : ::  ::.:..   .. ...:: .::.. .. . : :.:..:    :  ::.:. . ::..:::. :..:   .
PDEELNYTQLFAAGAFSGIFTTTVMAPGERIKCLLQIQQG-GNSPQKYSGMVDCAKQLYAEGGMRSIYKGAFATLLRDVPASGMYFLTYEYIQRALAPKA
       110       120       130       140        150       160       170       180       190

          190       200       210       220       230       240       250       260       270
P---------FVILMAGGLAGTFSWLVTFPLDVVKSRLQADGISGKPQYNGLIDCVRKSHAAEGWAFLSRGLASTLLRAFPMNAVCFLVVSYTMKLFDDP
          .  ..:::.::  .: . .: ::.:::::.    .   :   ::. :  :.     ::    : .:.. ..::::: ::.::. :.   ::...
GEQKDASIGLLGTIFAGGMAGIANWAIGMPADVLKSRLQTAPEGTYP--NGIRDVFRELMRREGPLALYKGVTPVMLRAFPANAACFIGVEVFMKFLNVV
200       210       220       230       240          250       260       270       280       290

280       290       300       310       320       330
KVSSVVEELGATAATVETPLLIVPTVVPQVPIATAPKLSVPAPAYNKRASHHDHDSHLLS

APGL
300
```

40.AGAP000079-PA BR9 X AGAP004718-PA 2L 35.043 1.56E-65

```
              10        20        30        40        50        60        70        80        90
      MERKRSIRRRQKPPFAERAKDHCRNFTAFMFSNVGIIFLVVLYMIAGAFMFIAIEGNEALERFAQIPFKRNETAMKLWQISCCEVNVFNKSVFE
                      :..:: ::.:.::: ..::.   :.: : ..::  :..:: .:       .  ..       :..  :  .::...   ..:.::.:...
MIRQRSSVRSRGSSSTTISDPREKVKDCCRKFVAFMCTQVGVGGLIVAYALVGAASFMSIETQEPNPLIEHVVTLRRNCAAELWDVTE-QLNLFNSSIWH
         10        20        30        40        50        60        70        80         90

  100       110       120       130       140       150       160       170       180       190
EKVGNEIRAYQEKIVLWARRGWQGSDITLESETPWSFSGAFLYSLTVITTIGYGNIVPRTEWGKIATIFYTIIGMPLFLLYLSNIGDILAKSFKWIYAKF
 ..    .. ::. ..   :::..:      . :  :.: .:... :.:.: :::::.:::: ::: ::..:. .:.::..::. :.: .::..:::.:. :
YEADLVLKRYQDDFAEAIRRGYDGR----SPEEVWNFPAALMFCLAVFTMIGYGNMVPRTAWGKGATVIYATFGIPLYILYFMNMGKVLASTFKWLYTWF
100       110       120           130       140       150       160       170       180       190

  200       210       220       230       240       250       260       270       280       290
CLCRVCPGVARRRAMRARRRARTEERDYSSEIGDNGFDTNTITTSNVDEEIEEEITAETNTVTVPITICIMIMIGYIFFGARLFADWENWDILDGSYFCF
  :               .   :.   . : : .:.                  :  . . :: : :. ..  ::   :. .::.::.:   ::..:::
HECS--------------HRSDEDGGLALEEGPGGL------------------APRKRIIVPTTACLWVITIYIATGTIMFAEWEKWTYLDSAYFCV
                 200       210                        220       230       240       250       260

  300       310          320       330       340       350       360       370
ISLSSIGFGDIVPGASL---QTKGDTKMEISFILCAVYLLLGMALIAMCFNLMQEQVIYKIRSLKKCVRGCFRCAKAVPVPMEEPES
 :: .::.::.::::..    :.    ::. :.:     ::.::::.:.:::. ::.:.:  :....:. .: :
TSLCKIGIGDLVPGANILDSQSGKPTKLVINF----VYMLLGMGLVAMCYILMREEVRIKMQEIKEDTRLCLEDLSSKFAKCCGTKDSQYYD
      270       280       290           300       310       320       330       340
```

The statistical note

1. Compute the p value for excess number. Based on the Figure 2a, the following code was executed in R environment.

```
excess <- matrix(c(2.44, 11.49, 2.07, 19,9,0), nrow = 2, byrow = T)
dimnames(excess) = list(c("ex", "ob"),c("xa", "aa","ax"))
chisq.test(excess, simulate.p.value = T, B = 1000000)
```

Note: The permutation test was designed firstly by Fisher, which is based on computationally intensive method to allow for any unclear distribution due to small sample size. The result is based on the approximation method of Monte Carlo simulation with simulate.p.value = T.

2. Compute p values for Table S3 (Baker and Papa's expression data) based on mean and standard error data from Vectorbase.

code: 1) design a function to compute summary test by using mean and standard errors:

```
t.test2 <- function(m1,m2,s1,s2,n1,n2,m0=0,equal.variance=FALSE)
{
    if( equal.variance==FALSE )
    {
        stderr <- sqrt( (s1^2/n1) + (s2^2/n2) )
        df <- ( (s1^2/n1 + s2^2/n2)^2 )/( (s1^2/n1)^2/(n1-1) + (s2^2/n2)^2/(n2-1) )
    } else
    {
        stderr <- sqrt( (1/n1 + 1/n2) * ((n1-1)*s1^2 + (n2-1)*s2^2)/(n1+n2-2) )
        df <- n1+n2-2
    }
    t <- (m1-m2-m0)/stderr
    res <- c(m1-m2, stderr, t, 2*pt(-abs(t),df))
    names(res) <- c("Difference of means", "Std Error", "t", "p-value")
    return(res)
}
```

2) input data and transfer standard error into standard deviations

For example,

```
x1=c(6.51,32.85,23.71,12.46,150.52,3.59,97.42,126.89,85.67,396.09,26.53,368.44,47.44,0.5
1,2.04,282.38,191.48,150.52,25.55,58.13,172.33,22.13,27.87,26.09,29.81,7.47,67.23,3.61,97
.14,19.74,10.38,7.51,38.88,4.81,0,3.59,70.57,3.65,3.7,26.53);
s1=c(0.74,9.34,7.85,2.68,15.84,1.3,9.11,7.55,9.69,44.25,4.01,46.89,2.21,0.13,0.43,27,50.82,
15.84,3.4,3.8,13.59,1.57,0.73,3.27,23.63,6.74,38.1,0.21,21.37,2.88,0.98,2.37,7.32,3.76,0,0.3
2,1.55,0.6,1.11,2.6)*sqrt(3);
x2=c(21.89,260.45,21.42,62.51,131.94,20.1,109.01,197.35,1159.71,539.81,90.79,1162.53,15
4.1,0.3,24.14,1504.47,156.24,131.94,394.14,143.34,302.74,180.07,66.38,77.94,21.81,24.13,
```

114.1,0.09,415.25,3.75,35.58,14.88,7.34,0.56,0,43.45,171.75,0.27,10.02,297.53);
s2=c(1.14,12.87,4.01,2.73,15.54,0.56,4.43,9.24,71.14,32.57,4.83,29.13,7.57,0.07,1.76,36.12,
5.93,15.54,14.33,3.97,15.13,24.42,3.46,6.1,14.49,17.3,87.35,0.09,10.74,0.44,2.96,2.49,6.16,
0.34,0,3.98,5.48,0.21,6.63,13.73)*sqrt(3);

3) compute the summary test for data

```
for(i in 1:length(x1)) {print(t.test2(x1[i], x2[i], s1[i], s2[i], 3, 3))}
```

3. Expression pattern across spermatogenesis stages

In R. I used the following code and changed very little for A>A type retrogene

```
a=read.table("sperm_rawexpr.txt",header=T)
df <- a %>%
   group_by(id, br,Branch,Pair,dr,gene, Stage) %>%
   summarise(mean = mean(log(Ex+1)),
                  std = sd(log(Ex +1))/sqrt(3))
a1=as.data.frame(df)
b=subset(a1, dr =="X>A")
ggplot(b, aes(x= Stage, y= mean, group=id, color= gene)) +
   geom_line() +
   geom_point()+
   geom_errorbar(aes(ymin=mean-std, ymax=mean+std), width=1,
                         position=position_dodge(0.05)) +facet_wrap(~Pair)
```

4. The test of gene enrichment based on KEGG and clusterProfiler. br17retrogene.txt is simply the gene-list including 19 retrogenes from BR1-7.

```
library(clusterProfiler)
a=read.table("br17retrogene.txt")
kk1 <- enrichKEGG(gene              = a$V1,
                         organism        = 'aga',
                         pvalueCutoff = 0.05)
head(kk1)
```
